# The impacts of physical activity on psychological and behavioral problems, and changes in physical activity, sleep and quality of life during the COVID-19 pandemic in preschoolers, children, and adolescents: A systematic review and meta-analysis

**DOI:** 10.3389/fped.2023.1015943

**Published:** 2023-03-13

**Authors:** Johnson C. Y. Pang, Eric L. S. Chan, Herman M. C. Lau, Kara K. L. Reeves, Tina H. Y. Chung, Heidi W. L. Hui, Alfred H. L. Leung, Allan C. L. Fu

**Affiliations:** ^1^ School of Health Sciences, Caritas Institute of HIgher Education, Tseung Kwan O, Hong Kong, Hong Kong SAR, China; ^2^ Discipline of Physiotherapy, Sydney School of Health Sciences, Faculty of Medicine and Health, The University of Sydney, Sydney, NSW, Australia; ^3^ Sydney Musculoskeletal Health, Faculty of Medicine and Health, The University of Sydney, NSW, Australia; ^4^ China Studies Centre, The University of Sydney, Sydney, NSW, Australia

**Keywords:** behavioral problems, COVID-19, physical activity, psychological problems, preschoolers, children and adolescents

## Abstract

**Background:**

The COVID-19 pandemic has greatly affected the level of physical activity (PA). However, little is known about its effect on health outcomes.

**Methods:**

Articles without language restrictions published from the database inception through March 16, 2022, were retrieved using the CINAHL Complete, Cochrane Library, EMBASE, Medline, PubMed, and PsycINFO databases. High-quality articles assessing the effect of PA on psychological and behavioral problems. Additionally, PA, QoL, and/or sleep problems before and during the pandemic were included. Articles without data regarding PA or involving non-general populations were excluded. The PRISMA and MOOSE guidelines were followed. Data quality of the selected articles was assessed using the Newcastle-Ottawa Scale and GRADE approach. Data were pooled using a random-effects model and sensitivity analysis if heterogenicity was high (*I*^2 ^≥ 50%). The relationship between PA and psychological and behavioral problems; and changes in PA, QoL, and sleeping patterns before and during the pandemic in preschoolers, children, and adolescents were investigated. A meta-analysis was conducted; odds ratios (ORs), mean differences (MD), and standardized MDs (SMDs) were calculated.

**Results:**

Thirty-four articles involving 66,857 participants were included. The results showed an overall significant protective effect between PA and psychological and/or behavioral problems (OR = 0.677; 95% CI = 0.630, 0.728; *p*-value <0.001; *I*^2^*^ ^*= 59.79%). This relationship was also significant in the subgroup analysis of children (OR = 0.690; 95% CI = 0.632, 0.752; *p*-value <0.001; *I*^2^*^ ^*= 58.93%) and adolescents (OR = 0.650; 95% CI = 0.570, 0.741; *p*-value <0.001; *I*^2^*^ ^*= 60.85%); however, no data on the relationship in preschoolers were collected. In addition, the overall time spent on PA significantly decreased by 23.2 min per day during the COVID-19 pandemic (95% CI = −13.5, −32.9; *p*-value <0.001; *I*^2^*^ ^*= 99.82%). Moreover, the results showed an overall significant decrease in QoL (SMD = −0.894, 95% CI = −1.180, −0.609, *p*-value <0.001, *I*^2^*^ ^*= 96.64%). However, there was no significant difference in sleep duration during the COVID-19 pandemic (MD = 0.01 h per day, 95% CI = −0.027, 0.225; *p*-value = 0.125; *I*^2^*^ ^*= 98.48%).

**Conclusion:**

During the pandemic, less PA was contributed to poor QoL and sleep quality. However, increases in PA are associated with reduced occurrences of psychological and behavioral problems. Implementing recovery plans to address the health effect of the pandemic is essential.

## 1. Introduction

On March 11, 2020, coronavirus disease (COVID-19) was declared a global pandemic by the World Health Organization (1). The long incubation period, high transmission rate, and ongoing viral mutation and evolution have posed great challenges to pandemic control (2). As of March 21, 2022, this highly contagious disease had affected 227 countries, resulting in 471,079,831 confirmed cases and 6,101,020 deaths globally (3). In addition to the loss of human life, the estimated loss of global economic output reached 2.96 trillion USD in 2020 (4). To contain the spread of COVID-19 and minimize such losses, countries around the world adopted preventive measures with varying levels of stringency (5), including the prohibition of mass gatherings, closure of schools and public places, physical distancing, or even lockdowns. These restrictive measures had a negative impact on the overall Physical activity (PA) levels in MET-min/weeks, as well as increased in sedentary behavior in young population (6). The measures had also caused changes in PA among children, and adolescents. For instance, a meta-analysis reported decrease in PA levels across all age groups during the pandemic (7). Among the total of 57 included studies, 16 of them reported solely on children and adolescents, and half of those indicated reduction in PA parameters (7). In addition, moderate-to-vigorous activities and step counts were significantly reduced in children and adolescents (7). Moreover, PA restrictions have negatively affected the psychological health of children and adolescents (8), further exacerbating their anxiety and depression. It is estimated that the pooled prevalence of clinically elevated depression and anxiety in children and adolescents increased to 25.2% and 20.5% respectively, which is twice the pre-pandemic estimate (9). Another study involving more than 59,000 young participants reported increased depressive symptoms and deteriorated psychological health during the pandemic (10). In addition to affecting psychological health, disturbing daily routines may induce changes in behavioral problems (11, 12), sleep quality (13, 14), and resulting quality of life (QoL) (15) in children and adolescents. Yet, a systematic review revealed a positive correlation between PA and psychological health in children and adolescent during the pandemic, indicating PA improves psychological health among them (8). In addition, a study with adult participants reported PA was associated with anxiety, emotional, psychological and social well-being, and sleep quality during the pandemic (16). And participants who were more active reported lower levels of anxiety, higher levels of well-beings and better sleep quality than those who were less active (16). Whereas the pandemic restrictive measures appeared to decrease PA participation, active lifestyle should still be encouraged as PA is beneficial to psychological health, sleep quality, and emotion and social well-being.

When faced with high psychological distress during the pandemic, increasing the level of PA is a coping strategy to mitigate the associated negative effects. In a study involving more than 1.2 million adult participants (17), those who exercised regularly experienced fewer days of poor psychological health than those who did not. Although the effects of increasing PA of preschoolers, children, and adolescents on psychological health (18) had been reported in several studies, the quality of those remains unclear. One systematic review (19) investigating the association between PA and psychological health in children and adolescents during the first year of the COVID-19 pandemic reported that an increasing level of PA is associated with fewer depressive symptoms, lower anxiety and stress, and an improved well-being and QoL. However, this association was analyzed only qualitatively. Furthermore, the study time frame was limited to the first year of the COVID-19 pandemic, and only four studies included preschoolers, children, and adolescents. Another systematic review (8) reported an association between PA and psychological health in preschoolers, children, and adolescents. Yet, this study only used PubMed as a search engine to identify potential articles, and no meta-analysis was conducted. Moreover, the quality of the selected articles was not assessed. Therefore, the selected articles may have had a risk of bias, potentially compromising the overall results. In addition, the extent of psychological health changes has not been reported; thus, the effect of PA changes cannot be quantified. The effect of PA on psychological and/or behavioral problems or sleep quality remains unknown in these reviews. As preschoolers, children and adolescents are considered a vulnerable population who are susceptible to mental health issues (18); their difficulties may be underestimated, and more investigations are needed in this group. Therefore, a systematic analysis of the aforementioned aspects is required.

This meta-analysis aimed to quantitatively analyze the relationship between PA changes and psychological problems in particular depression, anxiety, stress and mood disturbance and/or behavioral problems including irritability, peer problems, conduct problem, hyperactivity or inattention, and prosocial behavior in preschoolers, children and adolescents. The study also explored the changes of PA levels with the differences in sleeping patterns and QoL before and during the pandemic among those population. We hypothesized that increasing PA levels are associated with decreasing the occurrence of psychological and/or behavioral problems in preschoolers, children and adolescents. Additionally, we hypothesized there will be significant differences in PA levels, sleeping patterns and QoL before and during the pandemic in preschooler, children and adolescents. The null hypothesis was that there would be no relationship between PA and psychological and/or behavioral problems and no differences in PA levels, sleeping patterns and QoL among those population. This study may provide valuable references for resource allocation and the formulation of effective management policies to address the needs of PA interventions for preschoolers, children, and adolescents.

## 2. Methods

### 2.1. Search strategy and selection criteria

This meta-analysis adhered to the Preferred Reporting Items for Systematic Review and Meta-Analyses (PRISMA) (20) and Meta-Analysis of Observational Studies in Epidemiology (MOOSE) (21) guidelines. The protocol for this meta-analysis was published in the PROSPERO database (registration number: CRD42022309209). The current study was conducted in adherence with the published protocol. A comprehensive literature search of the CINAHL Complete, Cochrane Library, EMBASE, Medline, PubMed, and PsycINFO databases without language restrictions was conducted on March 16, 2022, for all relevant articles containing quantitative data. The reference lists of all relevant articles were also manually searched to minimize the possibility of missing data. For non-English articles, we initially used “Google Translate,” followed by consultation of professional translations by native speakers. The search history is presented in Supplementary Table S1, while the search terms are listed in Supplementary Table S2. In order to identify the relationship between PA and psychological and/or behavioral problems as well as the pattern of changes of PA levels with sleeping patterns and QoL, articles that either provide correlational quantitative data assessing the effect of changes in PA on psychological and/or behavioral problems during the COVID-19 pandemic, or mean changes in various parameters regarding to PA levels, sleeping patterns and/or QoL before and during the COVID-19 pandemic in preschoolers (age 0–5 years), children (age 6–11 years), and adolescents (age 12–18 years) were included. Moreover, only articles with a low risk of bias were included in order to provide rigorous findings. We excluded articles that involved participants who were not representative of the general preschool, child, and adolescent populations (e.g., articles that exclusively involved participants with pre-existing physiological or psychological health problems and athletes). Abstracts, editorial comments, and unpublished studies were also excluded.

### 2.2. Risk of bias and certainty assessment

The quality and certainty of the included articles were assessed by two independent reviewers (JP and KR) using the Newcastle-Ottawa Scale (NOS) (22) for cohort studies and the Grading of Recommendations Assessment, Development and Evaluation (GRADE) approach to assess the quality of evidence (23). The NOS has a maximum score of 9 points. The cut-off score for adequate quality in this meta-analysis was 7 points. Articles that scored 7–9, 4–6, and 0–3 points were considered to have a low, moderate, and high risk of bias, respectively (22). The GRADE includes four levels of certainty in evidence: very low, low, moderate, and high (23). The quality of evidence was applied to each outcome as this may vary across outcomes. A third reviewer resolved any disagreements regarding the scoring of the included articles.

### 2.3. Data extraction and statistical analysis

Based on the inclusion and exclusion criteria, the titles and abstracts of potential articles were screened by two independent reviewers, and the full texts of the remaining articles were evaluated. Disagreements were resolved by a third reviewer. Relevant data were extracted from the included articles by five reviewers using a standardized data extraction sheet. The variables are descripted as follow:

Relationship between PA and psychological and/or behavioral problems: quantitative data representing the relationship between PA and psychological and/or behavioral problems were retrieved. The impact of PA was measured by participants who were either being physically active, participated in moderate or high level of PA or indication of participation in PA during the pandemic on their occurrence of psychological problems, notably depression, anxiety, stress and mood disturbance and/or behavioral problems such as irritability, peer problems, peer problems, conduct problem, hyperactivity or inattention, and prosocial behavior. The participants’ levels of PA and their occurrence of psychological and/or behavioral problems were quantified by their responses in the selected parameters in the included articles.

PA levels, sleeping patterns and QoL: the differences in participants’ PA levels sleeping patterns including sleep duration and sleep quality, and QoL before and during the COVID-19 pandemic were extracted. Particularly, articles that reported the difference in time spent on PA and sleep duration were recorded to identify the mean time spent change in PA and sleep duration before and during the pandemic. For articles that did not provide changes in time spent in PA, and rather adopted other parameters, we provided standardized mean differences to identify the changes. Similarly, standardized mean differences were calculated for changes in sleep quality and QoL before and during the pandemic.

Additionally, confounding variables, including participants’ age, were assessed in a subgroup analysis. The authors of the selected articles would be contacted and any missing data were reported. Comprehensive Meta-Analysis version 3.0 (Biostat, Englewood, New Jersey, United States) was used for the statistical analysis. The targeted outcomes were presented as the mean difference (MD) with 95% confidence intervals (CIs) for data reported in hours per day and standardized MD (SMD) with 95% CIs for data reported with other measures. To identify the relationship between PA and psychological and/or behavioral changes, we generated pooled effects estimated as odd ratios (ORs) with 95% CIs. All *p*-values in this meta-analysis were two-tailed, and statistical significance was set at ≤0.05. The risk of heterogenicity was assessed by the *I*^2^ index, and a random-effects model was selected if the heterogeneity was ≥50%. The risk of publication bias was assessed by funnel plots and Egger’s test (24). An asymmetric plot and *p* ≤ 0.05 indicated the risk of publication bias. A sensitivity analysis was conducted to evaluate the robustness of the relationship (pooled ORs ratio) between PA and psychological and/or behavioral problems by removing each included study from the analysis one by one.

## 3. Results

### 3.1. Study characteristics

**Figure 1:**
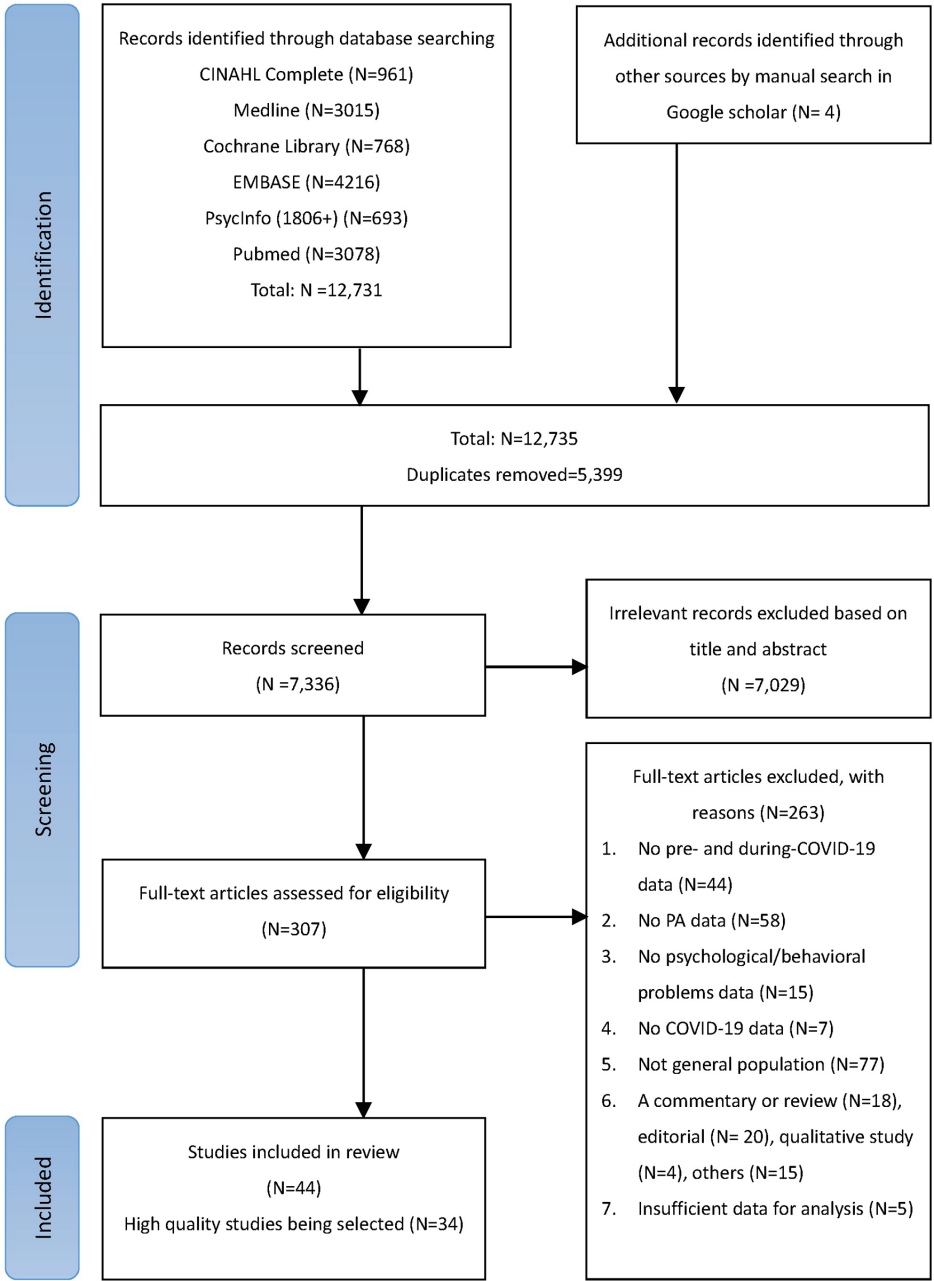
PRISMA flow chart of study selection in the systematic review.

In total, 12,735 potential articles were retrieved from the literature search. After removing duplicates and screening titles and abstracts, 307 full-text articles were reviewed, of which 263 articles were further removed, leaving 44 articles (11, 14, 23, 25–66). The PRISMA flowchart of the study selection is shown in Figure 1, and the reasons for exclusion are detailed in Supplementary Table S3. The authors of the selected articles were not contacted to obtain additional data, as this was not required in this meta-analysis.

The NOS scores of all included articles are documented in Supplementary Table S4. After excluding studies with a high-to-moderate risk of bias, 34 articles (25, 27, 29–34, 36–50, 53, 55, 56, 58, 61, 62, 64–66), comprising 66,857 participants and 23 different countries, were included in the analysis. The included articles had NOS scores between 7 and 8 out of 9 points and were classified as having a low risk of bias and had a rating of 4 under the modified Oxford Centre for Evidence-based Medicine (67). The certainty of the included studies was assessed by GRADE (Supplementary Table S5). The characteristics of the included articles are summarized in Table 1.

**Table 1:** Characteristics of included articles.

Authors	Country	Age mean (SD)	Age range	Sample size	Sampling timepoint (Before COVID-19)	Sampling timepoint (During COVID-19)
Abid et al. (2021)	Tunisia	8.66 (3.30)	5–12	100	Not reported	(Apr to May 2020)
Aguilar-Farias et al. (2020)	Chile	3.10 (1.38)	1–5	3,157	Not reported	(Mar to Apr 2020)
Alonso-Martínez et al. (2021)	Spain	4.29 (0.76)	4–6	425	(Sep to Dec 2019)	(Mar to Apr 2020)
Breidokienè et al. (2021)	Lithuania	9.65 (1.94)	6–14	306	Not reported	2020
Bringolf-Isler et al. (2021)	Switzerland	Not reported	5–11	1,712	(2014 and 2015)	2022
Brzęk et al. (2021)	Poland	Not reported	3–5	1,316	Not reported	(Apr to Nov 2020)
Burdzovic Andreas and Brunborg (2021)	Norway	Grade 11 (est. 16)	Not reported	2,536	2017	(Oct to Dec 2020)
Chen et al. (2021)	China	Not reported	11–20	13,440	(Feb 2020)	(Apr 2020)
Chen et al. (2022)	Sweden	13.60 (0.4)	Not reported	1,901	(Sep 2017 and Jan 2020)	(Feb and Nov 2020)
Dragun et al. (2020)	Croatia	17.00 (1.00)	Not reported	1,093	(2018 and 2019)	(May 2020)
Francisco et al. (2020)	Italy, Spain, Portugal	9.15 (4.27)	3–18	1,480	Not reported	(Mar to Apr 2020)
Ghanamah and Eghbaria-Ghanamah (2020)	Israeli	Not reported	5–11	382	Not reported	(Dec 2020)
Ghorbani et al. (2021)	Iran	16.28 (0.97)	15–17	136	Not reported	(Oct 2020 to Feb 2021)
Gibert et al. (2021)	USA	8.01 (1.75)	Not reported	144	Not reported	(May to Jul 2020)
Hossain et al. (2021)	Bangladesh	4.50 (0.15)	Not reported	65	(Mar to Jun 2019)	(May 2020)
Hyunshik et al. (2021)	Japan	3.60 (0.3)	3–5	591	(Oct 2019)	(Oct 2020)
Ishimoto et al. (2022)	Japan	Not reported	8–12	293	(Dec 2019)	(March 2020)
Jackson et al. (2021)	USA	13.84 (2.74)	10–18	624	Not reported	(Apr to Jun 2020)
Jauregui et al. (2021)	Mexico	3.30 (0.20)	1–5	631	Not reported	(Apr to Jul 2020)
Jester and Kang. (2021)	UK	16.64 (1.29)	15–18	55	Not reported	(Apr to Jun 2020)
Jovanović et al. (2021)	Croatia	12.72 (1.17)	10–15	1,370	Not reported	(May 2021)
Kang et al. (2021)	China	16.30 (1.30)	Not reported	4,898	Not reported	(Mar 2020)
Kuhn et al. (2022)	USA	Not reported	3–15	75	(Oct 2017 to Mar 2020)	(May to Jul 2020)
Laurier et al. (2021)	Canada	15.26 (1.46)	11–17	133	Not reported	(Jun to Aug 2020)
Lim et al. (2021)	Singapore	*Median (IQR) 8 (6–11)	3–16	593	Not reported	(Apr to Jun 2020)
Lu et al. (2021)	China	15.26 (0.46)	Not reported	965	Not reported	(May 2020)
Łuszczki et al. (2021)	Poland	10.51 (2.13)	6–15	1,016	(Feb to Mar 2020)	(Feb to Mar 2021)
McArthur et al. (2021)	Canada	Not reported	9–11	846	(2017–2019)	(May to Aug 2020)
Medrano et al. (2021)	Spain	12.10 (2.40)	8–16	404	(Sep to Dec 2019)	(Mar to Apr 2020)
Mzadi et. al. (2022)	Morocco	16.55 (0.96)	15–18	807	(2014–2015)	(Sep 2020 to Feb 2021)
Ren et al. (2021)	China	13.14	10–17	1,487	Not reported	(Apr 2020)
Wang et al. (2021)	China	9.10 (1.33), 13.90 (1,40)	6–11, 12–16	12,186	Not reported	(May to Jul 2020)
Wunsch et al. (2021)	Germany	10.36 (4.04)	4–17	1,711	Not reported	(Apr 2020)
Zhang et al. (2020)	China	11.63 (1.23)	9–14	9,979	Not reported	(Mar 2020)

* Only age median.

### 3.2. Outcomes

#### 3.2.1. Relationship between PA, and psychological and/or behavioral problems during the COVID-19 pandemic

Overall, 14 articles (29, 33, 39, 40, 43, 44, 48, 50, 55, 58, 61, 62, 64, 66) reported the relationship between PA and psychological and/or behavioral problems during the COVID-19 pandemic (Table 2). To measure the PA levels, ten studies (29, 33, 40, 43, 44, 50, 58, 62, 64, 66) used a self-designed questionnaire, three studies adopted a validated questionnaire including the International Physical Activity Questionnaire Short Form (IPAQ-SF) (48, 55) and Goldin-Shepard Leisure—The Physical Activity Questionnaire (61), and one study utilized an accelerometer (39). To identify the occurrence of psychological and/or behavioral problems, nine studies (29, 33, 40, 43, 44, 58, 62, 64, 66) used a self-designed questionnaire where 5 studies employed a validate questionnaire such as the simplified Chinese Profile of Mood Status (POMS) (48), Depression, Anxiety, Stress Scale-21 (DASS-21) (39), The Psychological Distress Index (50), 9-Items Patient Health Questionnaire (PHQ-9) (55) and Generalized Anxiety Disorder Scale (GAD-7) (55), and Brief Symptom Inventory (BSI) (61). Our results showed an overall significant protective effect between PA and psychological problems include depression, anxiety, stress and mood disturbance and/or behavioral problems such as irritability, peer problems, conduct problem, hyperactivity or inattention, and prosocial behavior (OR = 0.677; 95% CI = 0.630, 0.728; *p*-value <0.001; *I*^2^*^ ^*= 59.79%). This relationship was also significant in the subgroup analysis of children (OR = 0.690; 95% CI = 0.632, 0.752; *p*-value <0.001; *I*^2^*^ ^*= 58.93%) and adolescents (OR = 0.650; 95% CI = 0.570, 0.741; *p*-value <0.001; *I*^2^*^ ^*= 60.85%); however, no data on the relationship in preschoolers were collected. The funnel plot analysis is shown in Supplementary Figure S1.

**Table 2:** The relationship between PA and psychological and/or behavior problems.

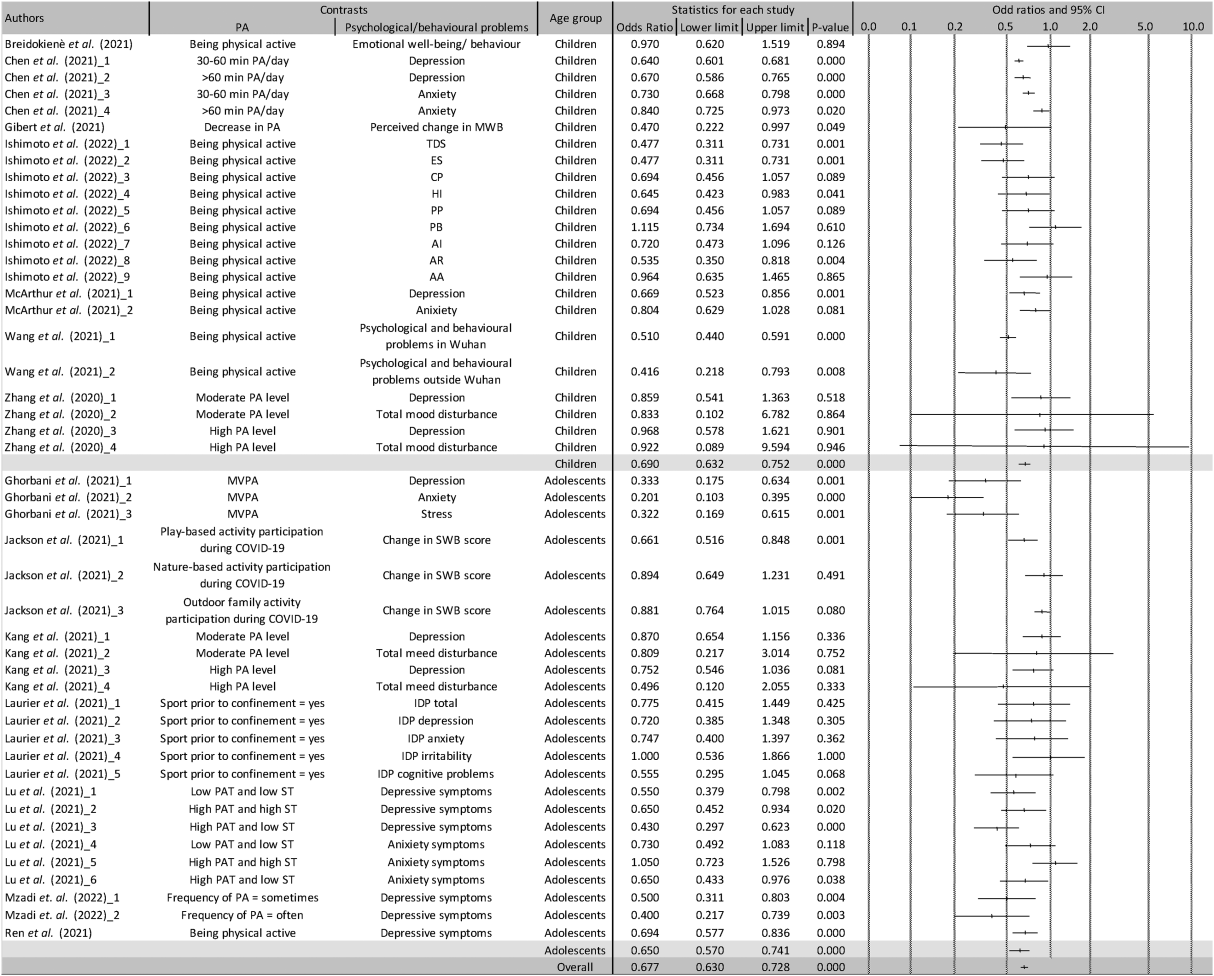

Key: The number after all the authors indicated different sets of data extracted for analysis in the outcomes accordingly. PA, physical activity; MWB, mental well-being; TDS, total difficulties score; ES, emotional symptoms; CP, conduct problems; HI, hyperactivity/inattention; PP, peer problems; PB, prosocial behavior; AI, anxiety related to infection; AR, anxiety related to returning to school; AA, anxiety related to academic delay; SWB, subjective well-being; IDP, psychological distress index; PAT, physical activity time; ST, sitting time.

#### 3.2.2. Changes in PA before and during the COVID-19 pandemic

The mean time changes in PA before and during the COVID-19 pandemic were identified in nine articles (27, 31, 38, 41, 42, 45, 49, 53) (Table 3A). Overall, the time spent on PA significantly decreased by 23.2 min per day during the COVID-19 pandemic (95% CI = −13.5, 32.9; *p*-value <0.001; *I*^2^*^ ^*= 99.82%). In the subgroup analysis, the time spent on PA also significantly decreased for preschoolers by 29.6 min per day (95% CI = −14.1, −45.1; *p*-value <0.001; *I*^2^*^ ^*= 99.89%), for children by 18.8 min per day (95% CI = −2.0, −35.4; *p*-value = 0.028; *I*^2^*^ ^*= 97.34%), and for adolescents by 19.4 min per day (95% CI = −1.0, −37.9; *p*-value = 0.039; *I*^2^*^ ^*= 84.54%). In addition, six articles (25, 32, 34, 44, 47, 65) reported changes in PA using various measurements (Table 3B). There was no significant difference in the pooled SMD in PA during the COVID-19 pandemic (SMD = −0.506, 95% CI = −1.070, 0.059; *p*-value = 0.079; *I*^2^*^ ^*= 99.51%).

**Table 3A:** Outcomes: the mean time change in PA before and during COVID-19.

Authors	Subgroup	Age group	Mean diff (mins/day)	Lower limit	Upper limit	*p*-value
Aguilar-Farias et al. (2020)	Not reported	Preschoolers	−47.4	−41.28	−53.52	<0.001
Alonso-Martínez et al. (2021)_1	MVPA	Preschoolers	−43.2	−2.88	−83.52	0.036
Alonso-Martínez et al. (2021)_2	Total PA	Preschoolers	−16.8	−0.84	−32.76	0.039
Brzęk et al. (2021)	Not reported	Preschoolers	−7.38	−5.58	−9.18	<0.001
Hossain et al. (2021)_1	MVPA	Preschoolers	−58.8	−48.60	−69	<0.001
Hossain et al. (2021)_2	Total PA	Preschoolers	−193.8	−174.66	−212.94	<0.001
Hyunshik et al. (2021)_1	Weekdays	Preschoolers	−4.8	−0.42	−9.18	0.031
Hyunshik et al. (2021)_2	Weekend	Preschoolers	−20.4	−15.18	−25.62	<0.001
Jáuregui et al. (2021)_1	MVPA	Preschoolers	−60.6	−59.22	−61.98	<0.001
Jáuregui et al. (2021)_2	Total PA	Preschoolers	−34.8	−33.90	−35.7	<0.001
Kuhn et al. (2022)_1	MVPA, weekday	Preschoolers	8.52	14.28	2.76	0.004
Kuhn et al. (2022)_2	MVPA, weekend	Preschoolers	30.36	53.82	6.9	0.011
Kuhn et al. (2022)_3	LPA, weekday	Preschoolers	0.48	0.84	0.12	0.014
Kuhn et al. (2022)_4	LPA, weekend	Preschoolers	28.8	49.74	7.86	0.007
		Preschoolers	−29.64	−14.10	−45.12	<0.001
Ghanamah and Eghbaria-Ghanamah (2020)	Not reported	Children	−51.6	−42.72	−60.48	<0.001
Kuhn et al. (2022)_5	MVPA, weekday	Children	0.96	1.62	0.3	0.004
Kuhn et al. (2022)_6	MVPA, weekend	Children	17.88	31.68	4.08	0.011
Kuhn et al. (2022)_7	LPA, weekday	Children	−32.1	−6.60	−57.6	0.014
Kuhn et al. (2022)_8	LPA, weekend	Children	−58.98	−16.26	−101.7	0.007
Lim et al. (2021)	Not reported	Children	−13.2	−8.64	−17.76	<0.001
		Children	−18.78	−2.04	−35.46	0.028
Kuhn et al. (2022)_9	MVPA, weekday	Adolescents	−15.3	−4.74	−25.86	0.004
Kuhn et al. (2022)_10	MVPA, weekend	Adolescents	−1.08	−0.24	−1.92	0.012
Kuhn et al. (2022)_11	LPA, weekday	Adolescents	−73.38	−14.22	−132.54	0.015
Kuhn et al. (2022)_12	LPA, weekend	Adolescents	−51.9	−13.62	−90.18	0.008
		Adolescents	−19.44	−1.02	−37.92	0.039
		Overall	−23.16	−13.50	−32.88	<0.001

**Table 3B:** Outcomes: the standardized mean difference in quality of life, PA and sleep quality.

Outcomes	Authors	Age	Measurements	Std diff in mean	Upper limit	Lower limit	*p*-value
Quality of life	Bringolf-Isler et al. (2021)	5–11	KINDL-R questionnaire	−1.106	1.207	1.004	<0.001
	Wunsch et al. (2021)_1	4–10	KIDSCREEN-10 index	−0.964	1.067	0.861	<0.001
	Wunsch et al. (2021)_2	11–17	KIDSCREEN-10 index	−0.612	0.717	0.507	<0.001
Overall				−0.894	1.180	0.609	<0.001
Physical activity	Abid et al. (2021)_1	8.66 (3.30)	Total PA score from PA Questionnaire	−0.695	0.980	0.409	<0.001
	Abid et al. (2021)_2	8.66 (3.30)	Leisure PA score from PA Questionnaire	−0.673	0.958	0.388	<0.001
	Abid et al. (2021)_3	8.66 (3.30)	Daily PA score from PA Questionnaire	−0.974	1.267	0.681	<0.001
	Burdzovic Andreas and Brunborg (2021)	Grade 11 (est. 16)	% of adolescent participated in organized sports	−0.205	0.331	0.078	0.002
	Chen et al. (2022)	13.50 (0.40)	PA 60 min/day (days/week)	0.000	0.097	0.097	1.000
	Jackson et al. (2021)	13.84 (2.74)	Outdoor Activity Score (Times per week from 5-point scale questionnaire)	−0.600	0.713	0.486	<0.001
	Jovanović et al. (2021)	12.72 (1.17)	MET-min/week	−2.164	2.259	2.070	<0.001
	Łuszczki et al. (2021)	10.51 (2.13)	PA 60 min/day (days/week)	−0.294	0.422	0.166	<0.001
	Wunsch et al. (2021)_1	11–17	Mo Mo Physical activity Questionnaire	0.169	0.067	0.270	0.001
	Wunsch et al. (2021)_2	4–10	Mo Mo Physical activity Questionnaire	0.361	0.271	0.451	<0.001
Overall				−0.506	1.070	0.059	0.079
Sleep quality	Abid et al. (2021)	8.66 (3.33)	Global PSQI score	−1.810	2.139	1.481	<0.001
	Aguilar-Farias et al. (2020)	3.10 (1.38)	Likert Scale score	−0.452	0.502	0.402	<0.001
	Alonso-Martínez et al. (2021)	4.28 (0.80)	Device-measured sleep efficiency (%)	−0.443	1.055	0.169	0.156
	Jaureguić et al. (2021)	3.30 (0.20)	Likert Scale score	−6.325	6.054	6.054	<0.001
	Łuszczki et al. (2021)	10.51 (2.13)	Four-point scale questionnaire	0.121	0.006	0.248	0.063
Overall				−1.785	3.392	0.177	0.030

#### 3.2.3. Sleeping patterns

The sleep duration before and during the COVID-19 pandemic was reported in 15 articles (25, 27, 34, 36–38, 41, 42, 45–47, 53, 56) (Table 3C). There was no significant difference in sleep duration during the COVID-19 pandemic (MD = 0.01 h per day, 95% CI = −0.027, 0.225; *p*-value = 0.125; *I*^2^*^ ^*= 98.48%). The subgroup analysis also showed no significant difference in sleep duration across age groups: preschoolers (MD = 0.01 h per day, 95% CI = −0.169, 0.190; *p*-value = 0.908; *I*^2^*^ ^*= 93.85%), children (MD = 0.17 h per day, 95% CI = −0.010, 0.357; *p*-value = 0.064; *I*^2^*^ ^*= 95.10%) and adolescents (MD = 0.36 h per day, 95% CI = −0.346, 1.074; *p*-value = 0.315; *I*^2^*^ ^*= 99.24%). However, five articles (25, 27, 44, 56) investigated changes in the sleep quality using different measurements (Table 3B). The pooled SMD indicated a significant decrease in sleep quality during the COVID-19 pandemic (SMD = −1.785, 95% CI = −3.392, −0.177; *p*-value = 0.030; *I*^2^*^ ^*= 99.80%).

**Table 3C:** Outcomes: the mean time change in sleep duration before and during COVID-19.

Authors	Subgroup	Age group	Mean diff (h/day)	Upper limit	Lower limit	*p*-value
Aguilar-Farias et al. (2020)	Not reported	Preschoolers	0.090	0.000	0.180	0.050
Alonso-Martínez et al. (2021)	Not reported	Preschoolers	0.110	−0.520	0.740	0.730
Hossain et al. (2021)	Not reported	Preschoolers	−0.210	−0.400	−0.020	0.030
Hyunshik et al. (2021)	Not reported	Preschoolers	0.070	−0.030	0.170	0.190
Jaureguić et al. (2021)	Not reported	Preschoolers	−0.200	−0.210	−0.190	<0.001
Lim et al. (2021)	Preschoolers	Preschoolers	0.340	0.083	0.597	0.010
		Preschoolers	0.011	−0.169	0.190	0.908
Abid et al. (2021)	Not reported	Children	0.010	−0.230	0.250	0.930
Francisco et al. (2020)_1	Total	Children	0.400	0.290	0.510	<0.001
Francisco et al. (2020)_2	Italy	Children	0.360	0.190	0.530	<0.001
Francisco et al. (2020)_3	Spain	Children	0.220	0.050	0.390	0.010
Francisco et al. (2020)_4	Portugal	Children	0.680	0.470	0.890	<0.001
Ghanamah and Eghbaria-Ghanamah (2021)	Not reported	Children	0.610	0.500	0.720	<0.001
Jovanović et al. (2021)	Not reported	Children	0.290	0.230	0.350	<0.001
Medrano et al. (2021)_1	Weekday	Children	0.000	−0.210	0.210	1.000
Medrano et al. (2021)_1	Weekend	Children	−0.200	−0.460	0.060	0.130
Lim et al. (2021)	Primary schoolers	Children	0.500	0.371	0.629	<0.001
Łuszczki et al. (2021)_1	Weekday	Children	−0.280	−0.454	−0.106	0.002
Łuszczki et al. (2021)_2	Weekend	Children	−0.590	−0.768	−0.412	<0.001
		Children	0.174	−0.010	0.357	0.064
Chen et al. (2022)_1	Non-school day	Adolescents	−0.260	−0.380	−0.140	<0.001
Chen et al. (2022)_2	School day	Adolescents	−0.610	−0.700	−0.520	<0.001
Dragun et al. (2020)_1	Non-working day	Adolescents	−0.500	−0.650	−0.350	<0.001
Dragun et al. (2020)_2	Working day	Adolescents	1.500	1.350	1.650	<0.001
Jester and Kang (2021)	Not reported	Adolescents	2.000	1.448	2.552	<0.001
Lim et al. (2021)	Secondary schoolers	Adolescents	0.200	−0.064	0.464	0.137
		Adolescents	0.364	−0.346	1.074	0.315
		Overall	0.099	−0.027	0.225	0.125

Key: The number after all the authors indicated different sets of data extracted for analysis in the outcomes accordingly. PA, physical activity; MVPA, moderate to vigorous physical activity; LPA, light physical activity; PSQI, Pittsburg Sleep Quality Index.

#### 3.2.4. QoL

Changes in QoL before and during the COVID-19 pandemic were revealed in two articles (30, 65) (Table 3B). The results showed an overall significant decrease in QoL (SMD = −0.894, 95% CI = −1.180, −0.609, *p*-value <0.001, *I*^2^*^ ^*= 96.64%).

### 3.3. Publication bias

Funnel plot analysis and Egger’s test (24) were performed to assess publication bias for the correlation between PA and psychological and behavioral problems. The shape of the funnel plots appeared fairly symmetrical, which suggested that the risk of publication bias was low. The findings from Egger’s test for odds ratios further confirmed that there was no publication bias (*t *= 0.48, *p *= 0.6312) (Supplementary Figure S1). Additionally, Egger’s test for the changes of PA, sleep duration, sleep quality and QoL before and after pandemic also confirmed that were no publication bias (*t *= 0.06, *p *= 0.118; *t *= 0.09, *p *= 0.464; *t *= 1.00, *p *= 0.195; *t *= 0.55, *p *= 0.680).

### 3.4. Quality of evidence

In our meta-analysis, only the cohort studies with the data comparing pre and during COVID-19 were included for the analysis of the changes in PA, sleep pattern and QoL. Additionally, those cohort studies reported the correlation of PA and psychological and behavior problems were selected. Overall quality of evidence for different outcomes and assessing the relationship between the changes in PA and psychological and behavioral changes was high to moderate. The heterogeneity of outcomes and small sample size downgraded the quality of evidence (Supplementary Table S5).

### 3.5. Sensitivity analysis

The pooled ORs of the relationship between PA and psychological and/or behavioral problems during the COVID-19 pandemic were not modified when removing each included study one by one. Further details are displayed in (Supplementary Table S6).

## 4. Discussion

This meta-analysis included more than 66,000 participants (preschoolers, children, and adolescents) from 23 countries. The findings support that increased PA (protective exposure) is associated with a reduced occurrence of psychological and behavioral problems during the COVID-19 pandemic for children (OR = 0.690, *p *< 0.001) and adolescents (OR = 0.650 *p *< 0.001). There was a significant decrease in the overall PA, sleep quality and QoL during the COVID-19 lockdown or constraints. However, the sleep duration was not significant change during the pandemic. Similar finding was reported by (68) that the duration of sleep time was not significant change (*p *= 0.11) though the sleep quality showed significant change that affect children’s psychological difficulties (*b *= 0.14, *t *= 6.87, *p *< 0.01). Besides, previous systematic review reported the magnitude of change in PA before and during the pandemic was difficult to be evaluated due to the limitation of lacking baseline data and heterogenicity of measurements (69). To our knowledge, this meta-analysis is the largest and most comprehensive evaluation of this relationship, with substantial evidence (overall OR = 0.677, *p *< 0.001), and quantified the changes of PA, sleep duration, sleep quality and QoL. Although the outcomes showed high heterogenicity due to the limitation of using varies measurements in the included studies, we have used random model to minimize the influence and we monitored the risk of publication bias. The current meta-analysis was based on the relative symmetrical shape of the funnel plot and the results from Egger’s test, and the quality of evidence was high to moderate by using a GRADE approach.

In addition, the association between the reduction in PA and psychological and behavioral problems during the COVID-19 pandemic remained when we performed subgroup analyses by age. Among the three groups, preschoolers were the most vulnerable, with significant reductions in PA (MD = −0.50, *p *< 0.001), followed by adolescents (MD = −0.32, *p *= 0.039) and children (MD = −0.31, *p *= 0.028). Policymakers and health-care professionals should provide more resources to cope with potential problems in these two groups. However, the change in sleep duration only increased by 0.001 h (*p *= 0.908). A significant overall decrease in PA may indicate that lifestyles become more sedentary (29, 66). In addition, the overall QoL decreased (SMD = −0.894, *p *< 0.001), and the sleep quality decreased (SMD = −1.785, *p *= 0.030), which may also be associated with adapted PA and a more sedentary lifestyle (29, 70). The potential adverse effects on the health of preschoolers, children, and adolescents are of great concern.

The relationship between interventions and the overall psychological well-being has not been established through meta-analyses. PA has inconsistent effects on mental health problems among younger and older children (9, 71); in addition, studies had highlighted the importance of prevention in mental health and behavioral problems in children and adolescents by limiting risk exposure and identifying high risk individuals early (72–75). Decrease in PA has shown to have detrimental effects in children and adolescents’ psychological and behavioral problem (76–78). Hence, a strategy for the early identification and prevention of decreased PA is essential.

Although the underlying mechanisms responsible for the effects of decreased PA on the psychological and behavioral status of preschoolers, children, and adolescents remain unclear, several hypotheses have been proposed. First the decreased participation in PA may elicit feelings of loneliness that negatively affect mental health (25). Second, restrictive measures during the pandemic may lead to social isolation and, hence, to psychological and behavioral problems (80). Those measures may also lead to less time participating in PA (81). Third, replacing healthy with unhealthy behaviors may have negative consequences for preschoolers, children, and adolescents. Given the limited evidence, more studies focusing on potential mechanisms between decreased PA and the psychological and behavioral status are needed to confirm these hypotheses. As the exact mechanisms are unknown, we investigated the relationship through a meta-analysis of valid studies focusing on preschoolers, children, and adolescents worldwide.

The strength of our meta-analysis lies in three key aspects. First, we included all available high-quality prospective studies based on the NOS. The quality of evidence was further analyzed by GRADE and the publication bias was evaluated by sensitivity analysis. Second, we quantified the amount of PA and assessed its association with psychological and behavioral problems in children and adolescents, thereby obtaining more meaningful information. Third, we did not exclude any non-English publications. As the pandemic is a worldwide health concern, it is necessary to include and analyze all scientific studies of different cultures in different countries.

### 4.1. Clinical implications

Previous studies reported that in reaction to the drastic decrease in PA among children and adolescents due to the policy restrictions in different countries, the change in healthy lifestyle triggered the changes in sleep quality and QoL (66, 82). In addition, prolonged stays at home increase the risk of inactivity that may contribute different psychological and behavior problems. However, there is no conclusion of the extent of the changes during the pandemic. Our study provides scientistic evidence to quantify the extent of changes in the PA, sleep pattern and QoL in the preschoolers, children and adolescents before and during the pandemic. Previous studies reported PA or physical exercise had little effect on psychological status (83–85). However, the current situation is changed during the pandemic. This meta-analysis was one of the first to reveal the association between PA and psychological and behavioural problems of preschoolers, children and adolescents of different countries in the context of the pandemic. Our findings provide robust evidence and theoretical guidance for related health promotion and psychological rehabilitation of preschoolers, children and adolescents in the epidemic or post-epidemic era. Our meta-analysis can also provide effective reference for relevant health promotion and policy implementation to help young people to recover from the adverse effects of the social isolation period in the epidemic.

### 4.2. Limitations

First, a limited amount of empirical data retrieved during the pandemic met the inclusion criteria. The aims were to identify the relationship of PA and psychological and/or behavioral problems and compare different aspects before and during the pandemic. Thus, only articles involving before and during pandemic comparisons or those reporting the relationship between PA and psychological and/or behavioral problems were included. Some articles that reported either only PA or only psychological or behavioral problems were excluded. Consequently, some data from cross-sectional investigations on PA and psychological or behavioral problems in some worldwide populations were excluded. The association between changes in PA and psychological and behavioral problems in preschoolers remains unknown as no empirical study has yet been conducted. This limitation may be caused by difficulties in assessing the psychological and behavioral changes of subjects who are too young to express themselves through questionnaires or formal interviews. Data from caregiver and parental observations may have risks of bias and inaccuracy. Further development of the direct silent observation of preschoolers is needed to solve this gap for future studies (86). Third, although we searched six scientific databases and expected the findings to cover nearly all relevant published articles without any language limitations, we cannot exclude the possibility that additional relevant articles may have been missed. However, in addition to manually searching other databases, the reference lists of all relevant articles were searched; thus, the number of missed articles was likely small and would have little effect on the analysis. Fourth, most studies included in the analysis collected information *via* self-report questionnaires and from different outcome measures, which could have led to errors in the measurement of PA. Consequently, the heterogeneity of the outcomes was high, which may indicate the high diversity of the effects with different level of PA. The variability derives mainly from the clinical heterogeneity that is difficult to minimize, because the selection of outcome measures is always diverse in empirical studies (87). The risk of methodological heterogeneity among the included studies was examined and minimized. Our findings support that the included studies share a similar cohort study design. The quality of these studies was assessed, and the risk of publication bias was evaluated. In addition, the statistical heterogeneity among the included study was controlled by using a random effect model as the heterogeneity *I*^2^ was ≥50% (88). Fifth, we could not entirely rule out the possibility that the caregivers or the children and/or adolescents themselves may have missed preclinical or undiagnosed psychological and behavioral problems. Based on our meta-analysis, further prospective studies are necessary to confirm the effect of decreased PA on psychological and behavioral problems in preschoolers, children, and adolescents. The potential reverse causality between decreased PA and psychological and behavioral problems should be identified early. Consequently, policymakers, health-care providers, and caregivers can minimize the risks of the adverse effects of decreased PA on society.

### 4.3. Conclusion

During the COVID-19 pandemic, less PA and longer screen times may induce a sedentary lifestyle. This may increase the risk of poor QoL and poor sleep quality. Increased PA is associated with fewer psychological and behavioral problems. Our results showed an overall significant protective effect between PA and psychological and/or behavioral problems. This relationship was also significant in terms of the subgroup analysis of children and adolescents. These findings may guide caregivers, health-care providers, and health-care policy makers in making recommendations and developing guidelines with respect to the degree of PA to help reduce the risk of psychological and behavioral problems at both the individual and population levels. More epidemiological studies with larger sample sizes and detailed quantifications of PA and psychological and behavioral problems will establish more precise information regarding this association. The influence of decreased PA on preschoolers’ psychological and behavioral problems remains uncertain due to the paucity of studies. The scientific gap can be resolved using sophisticated technology to provide accurate observations and assessments of preschoolers in the future.

## Data Availability

The datasets presented in this study can be found in online repositories. The names of the repository/repositories and accession number(s) can be found in the article/**Supplementary Material**.
