# Supplementary material for: The impacts of physical activity on psychological and behavioral problems, and changes in physical activity, sleep and quality of life during the COVID-19 pandemic in preschoolers, children, and adolescents: A systematic review and meta-analysis
Source: Front Pediatr. 2023 Mar 13;11:1015943. doi: 10.3389/fped.2023.1015943 (PMC10038232; doi:10.3389/fped.2023.1015943)
Supplement: Supplementary file 1 [file Table1.docx]

Supplementary Material

1. **Supplementary Figure**


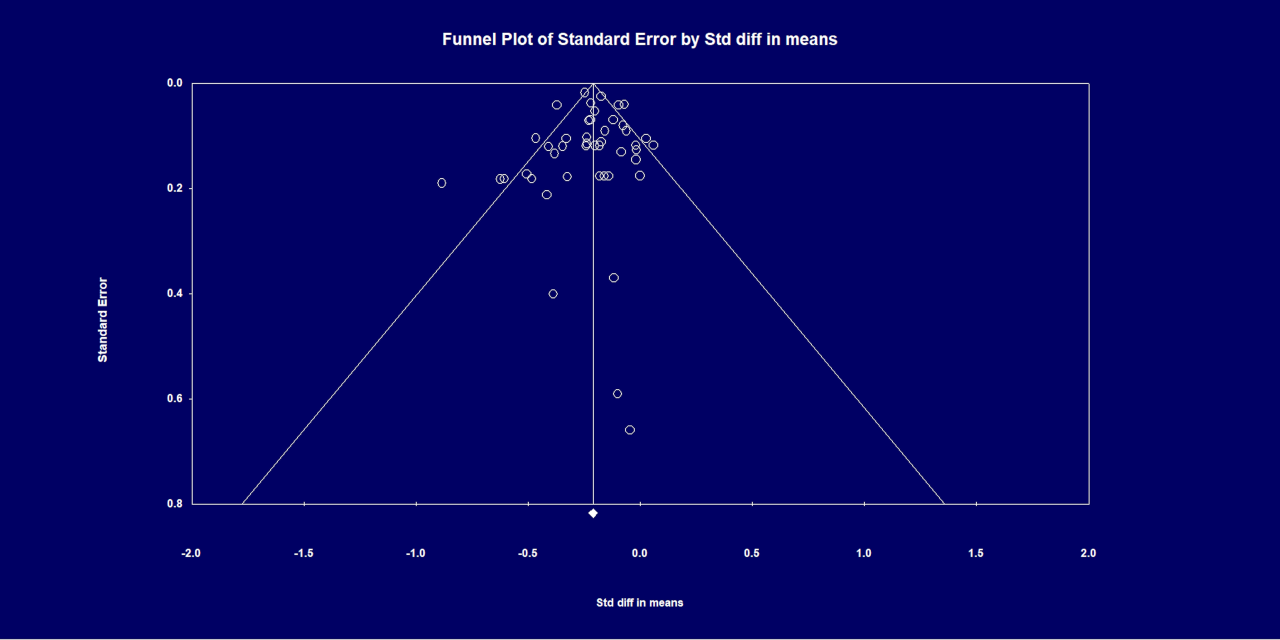


**Supplementary Figure 1. Funnel-plot of standard error by log odds ratio.**


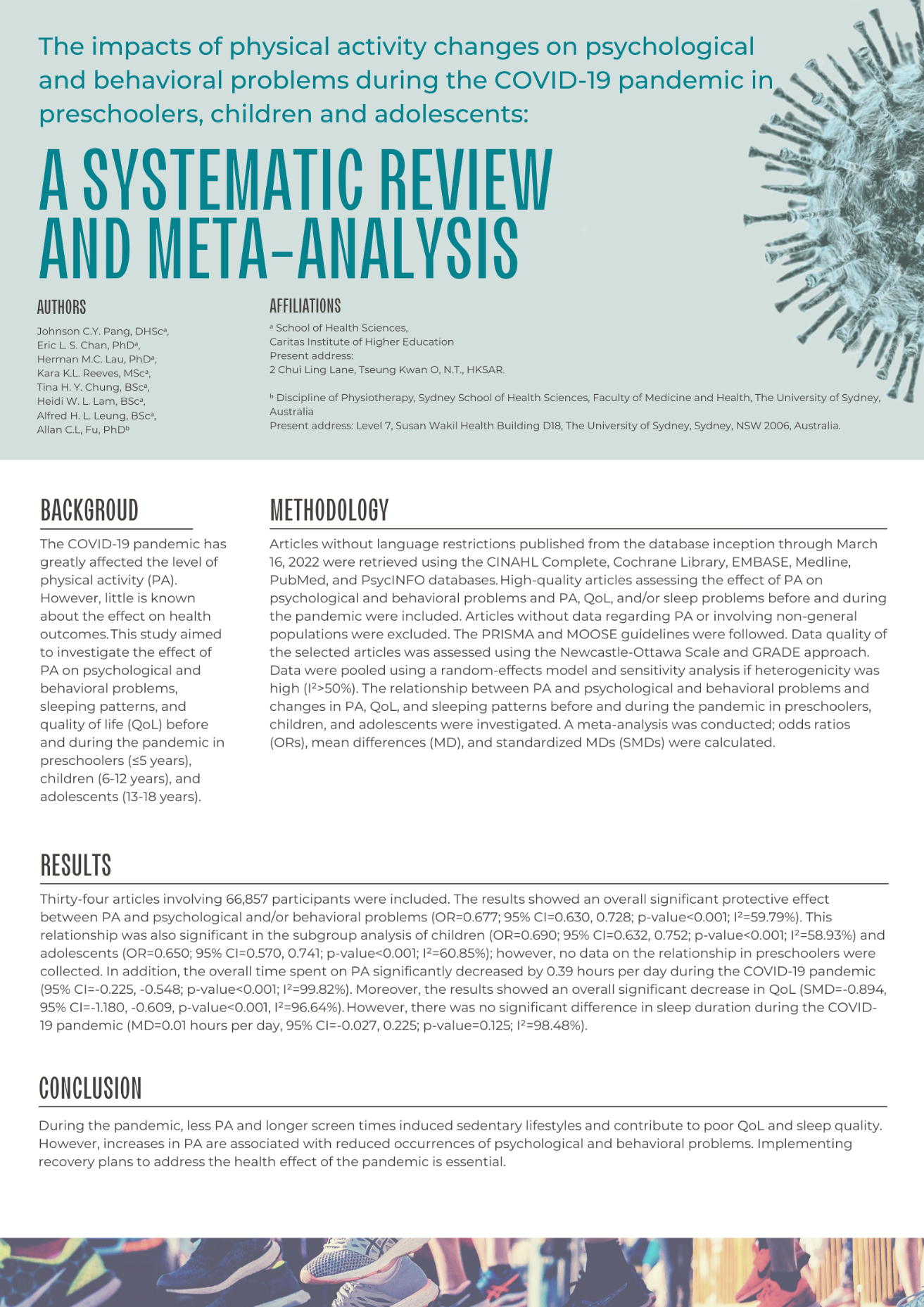


**Supplementary Figure 2. Poster abstract.**

1. **Supplementary Tables**

**Supplementary Table 1. The search terms used in the lectures search.**

| **Concepts** | **Search terms** |
| --- | --- |
| COVID-19 | “COVID” or “COVID-19” or “pandemic” or “coronavirus” or “2019-ncov” or “cov-19” |
| Physical activity changes | “physical activit*” or “physical activity change*” or “exercise*” or “fitness” or “physical exercise*” or “sport*” |
| Impacts | “quality of life” or “well being” or “well-being” or “health-related quality of life” or “playing behavior” or “sleep quality” or “quality of sleep” or “sleep problem*” or “sleep duration” or “sleep disorders” or “psychological stress” or “psychology*” or “psychological” or “mental health” or “depression” or “anxiety” or “psychological distress” or “psychological impact*” |

**Supplementary Table 2. Search history on 16th March 2022.**

| CLNAHL  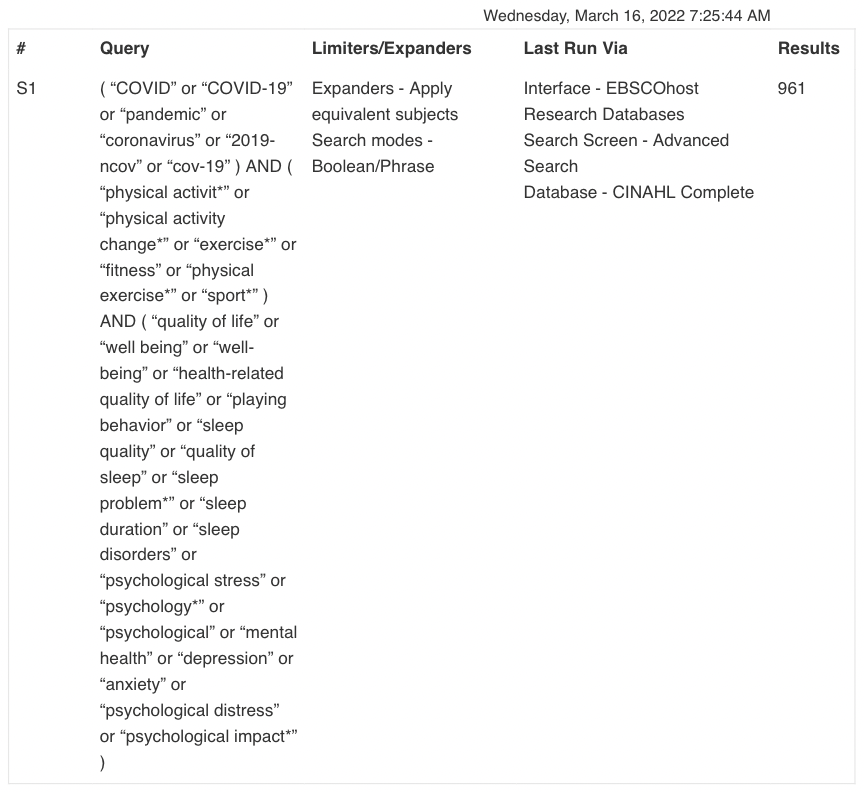 |
| --- |
| Cochrane Library  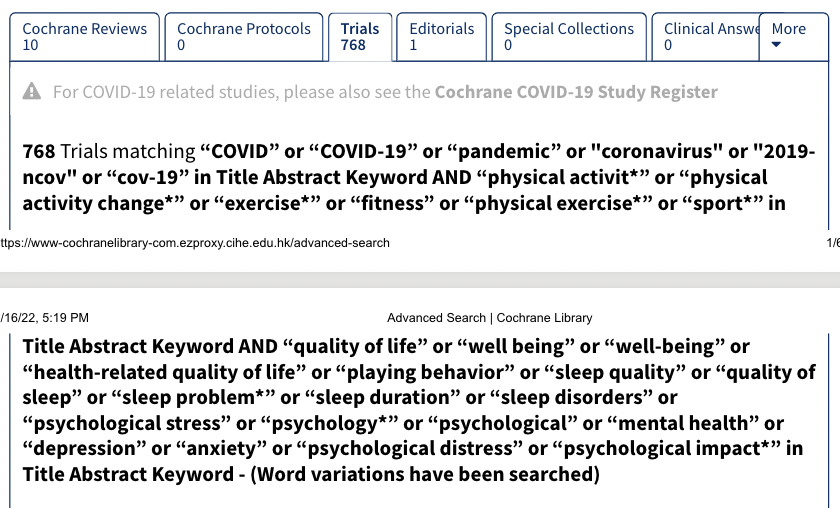 |
| Embase  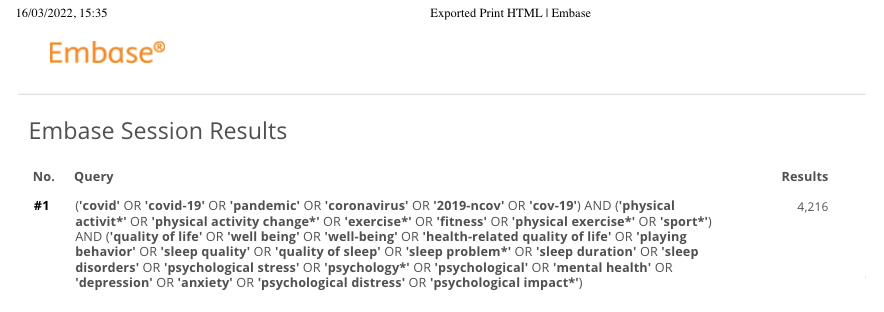 |
| Medline  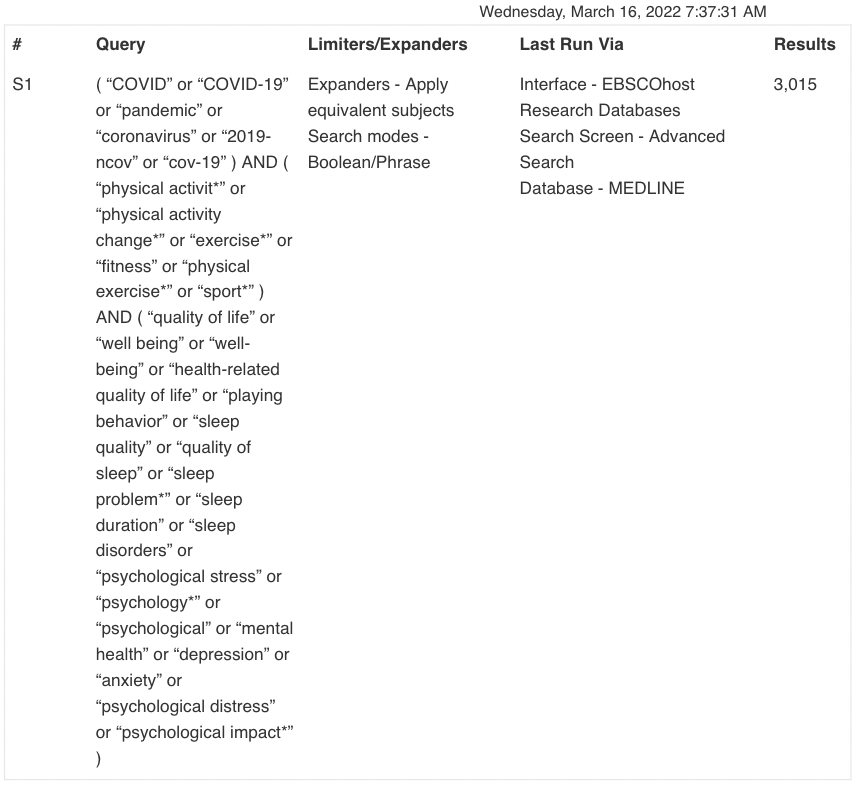 |
| PsycINFO  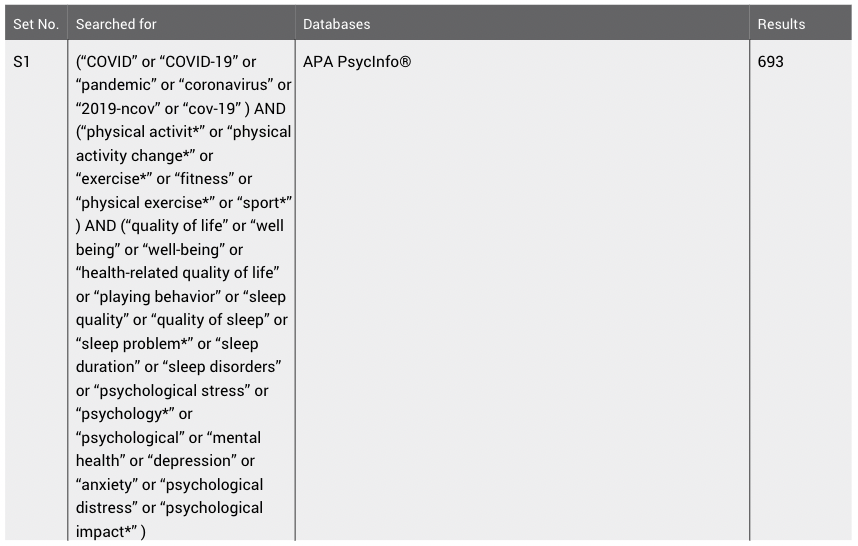 |
| PubMed  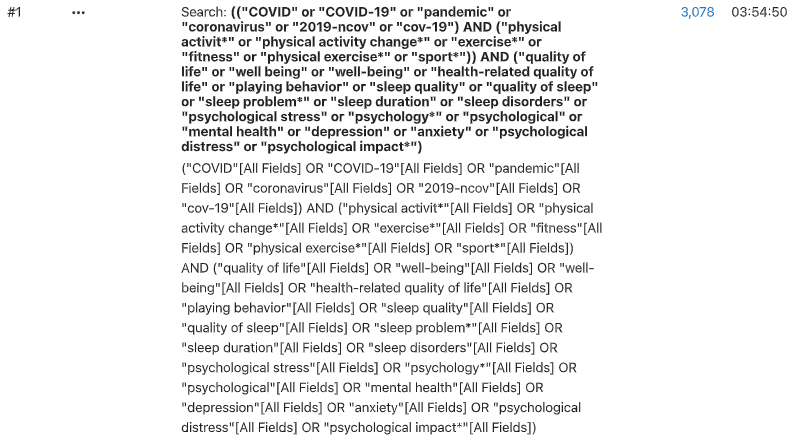 |

**Supplementary Table 3. Reasons for exclusion.**

| Reasons | Authors |
| --- | --- |
| No pre- and during- COVID-19 data or correlational data (N=44) | (Alves *et al.*, 2020), (Androutsos *et al.*, 2021), (Awais *et al.*, 2021), (Bates *et al.*, 2020), (Bösselmann *et al.*, 2021), (Chaturvedi *et al.*, 2021), (de Lannoy *et al.*, 2020), (Docimo *et al.*, 2021), (Dubuc *et al.*, 2021), (Dunton *et al.*, 2020), (Dzielska *et al.*, 2021), (Erades & Morales, 2020), (Gabriel *et al.*, 2020), (Hagen *et al.*, 2021), (Hosen *et al.*, 2021), (Ijaz & Ijaz, 2021), (Jolliff *et al.*, 2021), (S. J. Kim *et al.*, 2021), (S. H. Li *et al.*, 2021), (López-Bueno *et al.*, 2020), (José Francisco López-Gil *et al.*, 2021), (Lourenço *et al.*, 2021), (Mitra *et al.*, 2020), (Ng *et al.*, 2021), (Nie *et al.*, 2021), (O’rourke *et al.*, 2022), (Peralta *et al.*, 2021), (Pierce *et al.*, 2021), (Z. Qin *et al.*, 2021), (Reséndiz-Aparicio, 2021), (Sadeghipour *et al.*, 2021), (Saito *et al.*, 2021), (Salzano *et al.*, 2021), (Siachpazidou *et al.*, 2021), (Suhua *et al.*, 2021), (Szpunar *et al.*, 2021), (Szwarcwald *et al.*, 2021), (Tandon *et al.*, 2021), (Vuković *et al.*, 2021), (L. Wang, Y. Hao*, et al.*, 2021), (L. Wang, Y. Zhang*, et al.*, 2021), (P. Wang *et al.*, 2021), (M. Xiang *et al.*, 2020) |
| No physical activity data (N=58) | (Adu *et al.*, 2021), (Amjadi, 2021), (Anbarasu & Bhuvaneswari, 2020), (Azoulay *et al.*, 2021), (Bahatheg, 2021), (Begum *et al.*, 2020), (Berki & Pikó, 2021), (Browne *et al.*, 2021), (Campbell *et al.*, 2021), (Chen *et al.*, 2020), (Christakis, 2020), (Commodari & La Rosa, 2020), (Courtney *et al.*, 2020), (Crescentini *et al.*, 2020), (Cuschieri & Grech, 2020), (DiSabella *et al.*, 2021), (Efuribe *et al.*, 2020), (Esteves *et al.*, 2020), (Ezpeleta *et al.*, 2020), (Faigenbaum *et al.*, 2020), (Feng *et al.*, 2020), (Fernández Cruz *et al.*, 2020), (Fernández *et al.*, 2020), (Ferrante *et al.*, 2021), (Foley *et al.*, 2021), (Ghosh *et al.*, 2020), (Gupta *et al.*, 2021), (Gupta & Nebhinani, 2020), (Hamoda *et al.*, 2021), (Hester, 2021), (Idoiaga *et al.*, 2020), (Iozzi *et al.*, 2020), (Jolliff *et al.*, 2021), (Jurak *et al.*, 2021), (Kiss *et al.*, 2022), (Lessard & Puhl, 2021), (Liao *et al.*, 2021), (J. F. López-Gil *et al.*, 2021), (MacKenzie *et al.*, 2021), (McCluskey *et al.*, 2021), (Miller *et al.*, 2021), (Mingazova *et al.*, 2021), (Morgül *et al.*, 2020), (Muratori & Ciacchini, 2020), (Nakajima *et al.*, 2021), (J. Qin *et al.*, 2021), (Ramos-Álvarez *et al.*, 2021), (Rice & Sher, 2021), (Sarker *et al.*, 2021), (Schnaiderman *et al.*, 2021), (Slomski, 2021), (Surekha *et al.*, 2021), (Tardif-Grenier *et al.*, 2021), (Toppe *et al.*, 2021), (Tso *et al.*, 2022), (Zhai *et al.*, 2021), (Zhang *et al.*, 2021), (Zhu *et al.*, 2021) |
| No COVID-19 data (N=7) | (Linck, 2018), (Martinez *et al.*, 2019), (Mehdizadeh *et al.*, 2019), (Moore *et al.*, 2014), (Sedumedi *et al.*, 2021), (Simpson *et al.*, 2021), (Zhang *et al.*, 2011) |
| Not general population (N=77) | ("The Impact of COVID-19 on the Well-Being of Division III Student-Athletes," 2020), ("The Impact of COVID-19 Pandemic on High Performance Secondary School Student-Athletes," 2020), ("COVID-19 and its impact on student-athlete depression and anxiety: the return to campus," 2021), (Al Agha *et al.*, 2021), (Alves *et al.*, 2021), (Batalla-Gavalda *et al.*, 2021), (Berasategi Santxo *et al.*, 2021), (Biyik *et al.*, 2021), (Boukrim *et al.*, 2021), (Bucak *et al.*, 2021), (Bulut *et al.*, 2021), (Carroll *et al.*, 2020), (Chen *et al.*, 2021), (Cipolla *et al.*, 2021), (Denerel *et al.*, 2021), (Di Cagno *et al.*, 2020), (Ding & Yao, 2021), (Dun *et al.*, 2021), (Elbarbary *et al.*, 2021), (Elliott *et al.*, 2021), (Engels *et al.*, 2021), (Faraji *et al.*, 2021), (Farì *et al.*, 2021), (Gjaka *et al.*, 2021), (Gruba *et al.*, 2021), (José Puccinelli *et al.*, 2021), (Kalvin *et al.*, 2021), (Kerekes *et al.*, 2021), (Kuygun Karci & Arici Gurbuz, 2021), (Lange *et al.*, 2021), (Lee *et al.*, 2021), (Y. Li *et al.*, 2021), (Z. L. Li *et al.*, 2021), (Lin *et al.*, 2020), (Lindoso *et al.*, 2021), (Logan, Ciftci*, et al.*, 2021), (Logan, Kavaklioglu*, et al.*, 2021), (López-Aymes *et al.*, 2021), (Luo *et al.*, 2021), (Mc Guine *et al.*, 2021), (McCormack *et al.*, 2020), (McDonnell *et al.*, 2021), (T. McGuine *et al.*, 2021), (T. A. McGuine, K. M. Biese*, et al.*, 2021), (T. A. McGuine, K. Biese*, et al.*, 2021), (McGuine *et al.*, 2020), (Mohammadzadeh *et al.*, 2020), (Moore *et al.*, 2021), (Morales *et al.*, 2021), (Munasinghe *et al.*, 2020), (Muñoz-Fernández & Rodríguez-Meirinhos, 2021), (Neshteruk *et al.*, 2021), (O'Donoghue *et al.*, 2021), (Pietrabissa *et al.*, 2021), (Pietrobelli *et al.*, 2020), (Pigaiani *et al.*, 2020), (Pigeaud *et al.*, 2021), (Piña *et al.*, 2021), (Przybylski *et al.*, 2021), (Qi *et al.*, 2020), (Schlichtiger *et al.*, 2020), (Schmits *et al.*, 2021), (Sciberras *et al.*, 2022), (Shah *et al.*, 2021), (Sharpe *et al.*, 2021), (Shaun *et al.*, 2021), (Shepherd, Evans, Gupta, McDonough, Doyle-Baker, Belton, Karmali, Pawer, Hadly, Pike, Adams, Babul, Yeates*, et al.*, 2021), (Sutter *et al.*, 2021), (Thomas *et al.*, 2021), (Valadez *et al.*, 2020), (Valenzise *et al.*, 2021), (Vall-Roqué *et al.*, 2021), (Welling, Abawi, Van Den Eynde, Van Rossum, Halberstadt, Van Den Akker*, et al.*, 2021), (Welling, Abawi, Van Den Eynde, Van Rossum, Halberstadt, Brandsma*, et al.*, 2021), (Wright *et al.*, 2021), (Yuan *et al.*, 2021), (Zhou *et al.*, 2020) |
| Not a study (N=57) | A commentary or review (N=18): (Agarwal *et al.*, 2021), (Ali, 2021), (Cachón-Zagalaz *et al.*, 2020), (Canli *et al.*, 2021), (W. Li *et al.*, 2021), (Margaritis *et al.*, 2020), (Nobari *et al.*, 2021), (Okuyama *et al.*, 2021), (Oliva *et al.*, 2021), (O'Loughlin *et al.*, 2021), (Paterson *et al.*, 2021), (Racine *et al.*, 2020), (Samji *et al.*, 2021), (Singh *et al.*, 2021), (Spitzer, 2021), (Storz, 2020), (Ye, 2020), (Zhang, 2021)  Editorial (N=20): (Hageman, 2020), (Hageman, 2021), (Iqbal & Tayyab, 2021), (Leung *et al.*, 2021), (Mittal *et al.*, 2020), (Nyberg, 2021), (Owen & Bould, 2021), (Pagoto & Conroy, 2021), (Patra & Patro, 2021), (Patterson *et al.*, 2021), (Ramadhan *et al.*, 2020), (Rezaeipour, 2021), (Sharma *et al.*, 2021), (Shek, 2020), (Sinaei *et al.*, 2021), (Thakur *et al.*, 2020), (Tse & Kwan, 2021), (Vyjayanthi *et al.*, 2020), (Wang *et al.*, 2020), (Mi Xiang *et al.*, 2020)  Qualitative studies (N=4): (El-Osta *et al.*, 2021), (Lindsay *et al.*, 2021), (Lorch & Fuchs, 2020), (Shepherd, Evans, Gupta, McDonough, Doyle-Baker, Belton, Karmali, Pawer, Hadly, Pike, Adams, Babul, Emery*, et al.*, 2021)  Others (N=15): (Berasategi *et al.*, 2020), (Golberstein *et al.*, 2021), (Guan *et al.*, 2020), (Hoffman & Miller, 2020), (Jarnig *et al.*, 2021), (Khan, 2020), (Luijten *et al.*, 2019), (Milajerdi *et al.*, 2021), (O'Kane *et al.*, 2021), (Okely *et al.*, 2021), (Phd, 2021), (Salussolia *et al.*, 2021), (Singh & Balhara, 2021), (Weatherspoon, 2021), (Wickramasinghe, 2021) |
| Insufficient data for analysis (N=19) | (Al-Rahamneh *et al.*, 2021), (Guo *et al.*, 2021), (Susilowati *et al.*, 2021), (M. T. Wang *et al.*, 2021), (Bingham *et al.*, 2021), (Chaffee *et al.*, 2021), (S. Y. Kim *et al.*, 2021), (Lanza *et al.*, 2021), (Morres *et al.*, 2021), (Nct, 2021), (Ng *et al.*, 2020), (Pombo *et al.*, 2021), (Pombo *et al.*, 2020), (Sá *et al.*, 2020), (Shneor *et al.*, 2021), (Štveráková *et al.*, 2021), (Tornaghi *et al.*, 2021), (Ventura *et al.*, 2021), (Wahl-Alexander & Camic, 2021) |

**References of Supplementary Table 3.**

Adu, A. A., Toy, S. M., Pasifikus Christa Wijaya, R., & Kiling, I. Y. (2021). Development of information system-based policy for covid-19 affected students in the semi-arid area of indonesia [Article]. *Indian Journal of Forensic Medicine and Toxicology*, *15*(3), 2021-2029. <https://doi.org/10.37506/ijfmt.v15i3.15612>

Agarwal, V., Ganesh, L., & Sunitha, B. K. (2021). Impact of COVID-19 on the mental health among children in China with specific reference to emotional and behavioral disorders. *International Journal of Human Rights in Healthcare*, *14*(2), 182-188. <https://doi.org/http://dx.doi.org/10.1108/IJHRH-05-2020-0035>

Al Agha, A. E., Alharbi, R. S., Almohammadi, O. A., Yousef, S. Y., Sulimani, A. E., & Alaama, R. A. (2021). Impact of COVID-19 lockdown on glycemic control in children and adolescents. *Saudi Medical Journal*, *42*(1), 44-48. <https://doi.org/10.15537/smj.2021.1.25620>

Al-Rahamneh, H., Arafa, L., Al Orani, A., & Baqleh, R. (2021). Long-term psychological effects of covid-19 pandemic on children in jordan [Article]. *International Journal of Environmental Research and Public Health*, *18*(15). <https://doi.org/10.3390/ijerph18157795>

Ali, E. (2021). COVID-19, the Child, & Mental health: A Systematic Review [Conference Abstract]. *Annals of Neurology*, *90*(SUPPL 26), S116-S117. <https://doi.org/10.1002/ana.26177>

Alves, J. M., Yunker, A. G., DeFendis, A., Xiang, A. H., & Page, K. A. (2020). Associations between Affect, Physical Activity, and Anxiety Among US Children During COVID-19. *MedRxiv : the preprint server for health sciences*. <https://doi.org/10.1101/2020.10.20.20216424>

Alves, J. M., Yunker, A. G., DeFendis, A., Xiang, A. H., & Page, K. A. (2021). Prenatal exposure to gestational diabetes is associated with anxiety and physical inactivity in children during COVID-19 [Article]. *Clinical Obesity*, *11*(1). <https://doi.org/10.1111/cob.12422>

Amjadi, K. (2021). Exploring Factors That Influence Children’s Growth and Development During a Pandemic [Review]. *Global Pediatric Health*, *8*. <https://doi.org/10.1177/2333794X211042464>

Anbarasu, A., & Bhuvaneswari, M. (2020). COVID-19 pandemic and psychosocial problems in children and adolescents in vellore-district [Article]. *European Journal of Molecular and Clinical Medicine*, *7*(7), 334-339. <https://www.embase.com/search/results?subaction=viewrecord&id=L2010205562&from=export>

Androutsos, O., Perperidi, M., Georgiou, C., & Chouliaras, G. (2021). Lifestyle changes and determinants of children’s and adolescents’ body weight increase during the first COVID-19 lockdown in Greece: The COV-EAT study [Article]. *Nutrients*, *13*(3), 1-11. <https://doi.org/10.3390/nu13030930>

Awais, M. A., Chaudhery, M. M., Khan, M. S., Butt, A. U. A., Malik, A. R., Khan, M. N., Khalid, A., Mahmood, M., Afzal, M. T., & Waseem, M. H. (2021). Factors contributing to distress among school and college-going adolescents during COVID-19 Lockdown: A cross-sectional study conducted in Sibi Balochistan, Pakistan. *Journal of education and health promotion*, *10*, 317. <https://doi.org/10.4103/jehp.jehp_1313_20>

Azoulay, E., Yackobovitch-Gavan, M., Yaacov, H., Gilboa, I., Lopez, A., Sheppes, T., Waksman, Y., Lebenthal, Y., & Brener, A. (2021). Weight Status and Body Composition Dynamics in Children and Adolescents During the COVID-19 Pandemic [Article]. *Frontiers in Pediatrics*, *9*. <https://doi.org/10.3389/fped.2021.707773>

Bahatheg, R. O. (2021). Young Children's Nutrition During the COVID-19 Pandemic Lockdown: A Comparative Study. *Early Childhood Education Journal*, 1-9. <https://doi.org/10.1007/s10643-021-01192-3>

Batalla-Gavalda, A., Cecilia-Gallego, P., Revillas-Ortega, F., & Beltran-Garrido, J. V. (2021). Variations in the mood states during the different phases of COVID-19’s lockdown in young athletes [Article]. *International Journal of Environmental Research and Public Health*, *18*(17). <https://doi.org/10.3390/ijerph18179326>

Bates, L. C., Zieff, G., Stanford, K., Moore, J. B., Kerr, Z. Y., Hanson, E. D., Barone Gibbs, B., Kline, C. E., & Stoner, L. (2020). COVID-19 Impact on Behaviors across the 24-Hour Day in Children and Adolescents: Physical Activity, Sedentary Behavior, and Sleep. *Children (Basel, Switzerland)*, *7*(9). <https://doi.org/10.3390/children7090138>

Begum, A., Sangeetha, S., & Sridevi, G. (2020). Awareness of stress among children during lockdown-a survey [Article]. *International Journal of Pharmaceutical Research*, *12*, 2082-2092. <https://doi.org/10.31838/ijpr/2020.SP2.223>

Berasategi, N., Idoiaga, N., Dosil, M., & Eiguren, A. (2020). Design and Validation of a Scale for Measuring Well-Being of Children in Lockdown (WCL). *Frontiers in Psychology*, *11*, 2225. <https://doi.org/10.3389/fpsyg.2020.02225>

Berasategi Santxo, N., Idoiaga Mondragon, N., Ozamiz-Etxebarria, N., & Dosil-Santamaria, M. (2021). Well-Being of Adolescents in De-Escalation Situation: Physical, Emotional, Social, and Academic Impact. *Frontiers in Psychology*, *12*, 646027. <https://doi.org/10.3389/fpsyg.2021.646027>

Berki, T., & Pikó, B. F. (2021). SEDENTARY LIFESTYLE MAY CONTRIBUTE TO THE RISK OF DEPRESSION DURING THE COVID-19 PANDEMIC: A Snapshot of Hungarian Adolescents [Article]. *European Journal of Mental Health*, *16*(2), 99-119. <https://doi.org/10.5708/EJMH.16.2021.2.5>

Bingham, D. D., Daly-Smith, A., Hall, J., Seims, A., Dogra, S. A., Fairclough, S. J., Ajebon, M., Kelly, B., Hou, B., Shire, K. A., Crossley, K. L., Mon-Williams, M., Wright, J., Pickett, K., McEachan, R., Dickerson, J., & Barber, S. E. (2021). Covid-19 lockdown: Ethnic differences in children’s self-reported physical activity and the importance of leaving the home environment; a longitudinal and cross-sectional study from the Born in Bradford birth cohort study [Article]. *International Journal of Behavioral Nutrition and Physical Activity*, *18*(1). <https://doi.org/10.1186/s12966-021-01183-y>

Biyik, K. S., Özal, C., Tunçdemir, M., Ünes, S., Delioğlu, K., & Günel, M. K. (2021). The functional health status of children with cerebral palsy during the COVID-19 pandemic stay-at-home period: A parental perspective [Article]. *Turkish Journal of Pediatrics*, *63*(2), 223-236. <https://doi.org/10.24953/turkjped.2021.02.006>

Bösselmann, V., Amatriain-Fernández, S., Gronwald, T., Murillo-Rodríguez, E., Machado, S., & Budde, H. (2021). Physical Activity, Boredom and Fear of COVID-19 Among Adolescents in Germany. *Frontiers in Psychology*, *12*, 624206. <https://doi.org/10.3389/fpsyg.2021.624206>

Boukrim, M., Obtel, M., Kasouati, J., Achbani, A., & Razine, R. (2021). COVID-19 and confinement: Effect on weight load, physical activity and eating behavior of higher education students in southern Morocco [Article]. *Annals of Global Health*, *87*(1), 1-11. <https://doi.org/10.5334/aogh.3144>

Browne, N. T., Snethen, J. A., Greenberg, C. S., Frenn, M., Kilanowski, J. F., Gance-Cleveland, B., Burke, P. J., & Lewandowski, L. (2021). When Pandemics Collide: The Impact of COVID-19 on Childhood Obesity. *Journal of Pediatric Nursing*, *56*, 90-98. <https://doi.org/10.1016/j.pedn.2020.11.004>

Bucak, I. H., Almis, H., Tasar, S. O., Uygun, H., & Turgut, M. (2021). Have the sleep habits in children of health workers been more affected during the COVID-19 pandemic? *Sleep Medicine*, *83*, 235-240. <https://doi.org/10.1016/j.sleep.2021.05.003>

Bulut, N. S., Yorguner, N., & Akvardar, Y. (2021). Impact of covid-19 on the life of higher-education students in İstanbul: Relationship between social support, health-risk behaviors, and mental/academic well-being [Article]. *Anadolu Psikiyatri Dergisi*, *22*(6), 291-300. <https://doi.org/10.5152/alphapsychiatry.2021.21319>

Cachón-Zagalaz, J., Sánchez-Zafra, M., Sanabrias-Moreno, D., González-Valero, G., Lara-Sánchez, A. J., & Zagalaz-Sánchez, M. L. (2020). Systematic Review of the Literature About the Effects of the COVID-19 Pandemic on the Lives of School Children. *Frontiers in Psychology*, *11*, 569348. <https://doi.org/10.3389/fpsyg.2020.569348>

Campbell, K., Weingart, R., Ashta, J., Cronin, T., & Gazmararian, J. (2021). COVID-19 Knowledge and Behavior Change among High School Students in Semi-Rural Georgia [Article]. *The Journal of school health*, *91*(7), 526-534. <https://doi.org/10.1111/josh.13029>

Canli, M., ÖZÜDoĞRu, A., & Kara, E. (2021). COVID-19 Döneminde Çocuklarda Fiziksel Aktivite: Sistematik Derleme. *Turkiye Klinikleri Journal of Sports Sciences*, *13*(2), 312-317. <https://doi.org/10.5336/sportsci.2020-79699>

Carroll, N., Sadowski, A., Laila, A., Hruska, V., Nixon, M., Ma, D. W. L., Haines, J., & On Behalf Of The Guelph Family Health, S. (2020). The Impact of COVID-19 on Health Behavior, Stress, Financial and Food Security among Middle to High Income Canadian Families with Young Children. *Nutrients*, *12*(8). <https://doi.org/10.3390/nu12082352>

Chaffee, B. W., Cheng, J., Couch, E. T., Hoeft, K. S., & Halpern-Felsher, B. (2021). Adolescents' Substance Use and Physical Activity Before and During the COVID-19 Pandemic. *JAMA Pediatrics*, *175*(7), 715-722. <https://doi.org/10.1001/jamapediatrics.2021.0541>

Chaturvedi, K., Vishwakarma, D. K., & Singh, N. (2021). COVID-19 and its impact on education, social life and mental health of students: A survey. *Children & Youth Services Review*, *121*, N.PAG-N.PAG. <https://doi.org/10.1016/j.childyouth.2020.105866>

Chen, F., Zheng, D., Liu, J., Gong, Y., Guan, Z., & Lou, D. (2020). Depression and anxiety among adolescents during COVID-19: A cross-sectional study [Letter]. *Brain, Behavior, and Immunity*, *88*, 36-38. <https://doi.org/10.1016/j.bbi.2020.05.061>

Chen, J., Sang, G., Zhang, Y., & Jiang, A. (2021). Intervention effect of the integration model on negative emotions of adolescents during the outbreak of Corona Virus Disease 2019 [Article]. *Psychiatria Danubina*, *33*(1), 86-94. <https://doi.org/10.24869/PSYD.2021.86>

Christakis, D. A. (2020). Pediatrics and COVID-19 [Note]. *JAMA - Journal of the American Medical Association*, *324*(12), 1147-1148. <https://doi.org/10.1001/jama.2020.14297>

Cipolla, C., Curatola, A., Ferretti, S., Giugno, G., Condemi, C., Delogu, A. B., Birritella, L., & Lazzareschi, I. (2021). Eating habits and lifestyle in children with obesity during the covid19 lockdown: A survey in an italian center [Article]. *Acta Biomedica*, *92*(2). <https://doi.org/10.23750/abm.v92i2.10912>

Commodari, E., & La Rosa, V. L. (2020). Adolescents in Quarantine During COVID-19 Pandemic in Italy: Perceived Health Risk, Beliefs, Psychological Experiences and Expectations for the Future. *Frontiers in Psychology*, *11*, 559951. <https://doi.org/10.3389/fpsyg.2020.559951>

Courtney, D., Watson, P., Battaglia, M., Mulsant, B. H., & Szatmari, P. (2020). COVID-19 Impacts on Child and Youth Anxiety and Depression: Challenges and Opportunities [Note]. *Canadian Journal of Psychiatry*, *65*(10), 688-691. <https://doi.org/10.1177/0706743720935646>

COVID-19 and its impact on student-athlete depression and anxiety: the return to campus. (2021). *Sport Journal*, N.PAG-N.PAG. <https://search.ebscohost.com/login.aspx?direct=true&AuthType=sso&db=ccm&AN=153294375&site=ehost-live&custid=s3890005>

Crescentini, C., Feruglio, S., Matiz, A., Paschetto, A., Vidal, E., Cogo, P., & Fabbro, F. (2020). Stuck Outside and Inside: An Exploratory Study on the Effects of the COVID-19 Outbreak on Italian Parents and Children's Internalizing Symptoms. *Frontiers in Psychology*, *11*, 586074. <https://doi.org/10.3389/fpsyg.2020.586074>

Cuschieri, S., & Grech, S. (2020). COVID-19: a one-way ticket to a global childhood obesity crisis? [Letter]. *Journal of Diabetes and Metabolic Disorders*, *19*(2), 2027-2030. <https://doi.org/10.1007/s40200-020-00682-2>

de Lannoy, L., Rhodes, R. E., Moore, S. A., Faulkner, G., & Tremblay, M. S. (2020). Regional differences in access to the outdoors and outdoor play of Canadian children and youth during the COVID-19 outbreak [Article]. *Canadian journal of public health = Revue canadienne de sante publique*, *111*(6), 988-994. <https://doi.org/10.17269/s41997-020-00412-4>

Denerel, N., Köyağasıoglu, O., Şenışık, S., Çiğdem, S., & Tunç, S. (2021). Effects of Long-Duration Home Isolation Linked to the COVID-19 Pandemic on Mental Health of Adolescent Athletes. *Pediatric Exercise Science*, *33*(4), 170-176. <https://doi.org/10.1123/pes.2020-0164>

Di Cagno, A., Buonsenso, A., Baralla, F., Grazioli, E., Di Martino, G., Lecce, E., Calcagno, G., & Fiorilli, G. (2020). Psychological impact of the quarantine-induced stress during the coronavirus (COVID-19) outbreak among Italian athletes [Article]. *International Journal of Environmental Research and Public Health*, *17*(23), 1-13. <https://doi.org/10.3390/ijerph17238867>

Ding, X., & Yao, J. (2021). Peer education intervention on adolescents' anxiety, depression, and sleep disorder during the covid-19 pandemic [Article]. *Psychiatria Danubina*, *32*(3-4), 527-535. <https://doi.org/10.24869/PSYD.2020.527>

DiSabella, M., Pierce, E. L., Ratnaseelan, A., McCracken, E., Langdon, R., Fletcher, A., Strelzik, J., & Borner, K. (2021). Pediatric headache experience during the COVID-19 pandemic [Conference Abstract]. *Headache*, *61*(SUPPL 1), 166. <https://doi.org/10.1111/head.14130>

Docimo, R., Costacurta, M., Gualtieri, P., Pujia, A., Leggeri, C., Attinà, A., Cinelli, G., Giannattasio, S., Rampello, T., & Di Renzo, L. (2021). Cariogenic risk and COVID-19 lockdown in a paediatric population [Article]. *International Journal of Environmental Research and Public Health*, *18*(14). <https://doi.org/10.3390/ijerph18147558>

Dubuc, M.-M., Berrigan, F., Goudreault, M., Beaudoin, S., & Turcotte, S. (2021). COVID-19 Impact on Adolescent 24 h Movement Behaviors. *International Journal of Environmental Research and Public Health*, *18*(17). <https://doi.org/10.3390/ijerph18179256>

Dun, Y., Ripley-Gonzalez, J. W., Zhou, N., Li, Q., Chen, M., Hu, Z., Zhang, W., Thomas, R. J., Olson, T. P., Liu, J., Dong, Y., & Liu, S. (2021). The association between prior physical fitness and depression in young adults during the COVID-19 pandemic-a crosssectional, retrospective study [Article]. *PeerJ*, *9*. <https://doi.org/10.7717/peerj.11091>

Dunton, G. F., Do, B., & Wang, S. D. (2020). Early effects of the COVID-19 pandemic on physical activity and sedentary behavior in children living in the U.S [Article]. *BMC Public Health*, *20*(1), 1351. <https://doi.org/10.1186/s12889-020-09429-3>

Dzielska, A. M., Nałȩcz, H., Kleszczewska, D., & Mazur, J. (2021). Consequences of the COVID-19 pandemic on adolescents' health and health behaviour [Conference Abstract]. *Cogent Medicine*, *8*. <https://doi.org/10.1080/2331205X.2021.2002558>

Efuribe, C., Barre-Hemingway, M., Vaghefi, E., & Suleiman, A. B. (2020). Coping with the COVID-19 crisis: A call for youth engagement and the inclusion of young people in matters that affect their lives. *Journal of Adolescent Health*, *67*(1), 16-17. <https://doi.org/http://dx.doi.org/10.1016/j.jadohealth.2020.04.009>

El-Osta, A., El-Osta, A., Alaa, A., Webber, I., Riboli Sasco, E., Bagkeris, E., Millar, H., Vidal-Hall, C., & Majeed, A. (2021). How is the COVID-19 lockdown impacting the mental health of parents of school-age children in the UK? A cross-sectional online survey [Review]. *BMJ Open*, *11*(5). <https://doi.org/10.1136/bmjopen-2020-043397>

Elbarbary, N. S., dos Santos, T. J., de Beaufort, C., Wiltshire, E., Pulungan, A., & Scaramuzza, A. E. (2021). The Challenges of Managing Pediatric Diabetes and Other Endocrine Disorders During the COVID-19 Pandemic: Results From an International Cross-Sectional Electronic Survey [Article]. *Frontiers in Endocrinology*, *12*. <https://doi.org/10.3389/fendo.2021.735554>

Elliott, S., Drummond, M. J., Prichard, I., Eime, R., Drummond, C., & Mason, R. (2021). Understanding the impact of COVID-19 on youth sport in Australia and consequences for future participation and retention [Article]. *BMC Public Health*, *21*(1), 448. <https://doi.org/10.1186/s12889-021-10505-5>

Engels, E. S., Mutz, M., Demetriou, Y., & Reimers, A. K. (2021). Levels of physical activity in four domains and affective wellbeing before and during the Covid-19 pandemic. *Archives of Public Health*, *79*(1), 1-9. <https://doi.org/10.1186/s13690-021-00651-y>

Erades, N., & Morales, A. (2020). Impacto psicológico del confinamiento por la COVID-19 en niños españoles: un estudio transversal [Confinement by COVID-19 and associated stress can affect children’s well-being]. *Revista de Psicología Clínica con Niños y Adolescentes*, *7*(3), 27-34. <https://www.proquest.com/scholarly-journals/impacto-psicol>ógico-del-confinamiento-por-la/docview/2501282555/se-2?accountid=164166

http://sfx.csids.edu.hk/cihe??url_ver=Z39.88-2004&rft_val_fmt=info:ofi/fmt:kev:mtx:journal&genre=article&sid=ProQ:ProQ%3Apsycinfo&atitle=Impacto+psicol%26oacute%3Bgico+del+confinamiento+por+la+COVID-19+en+ni%26ntilde%3Bos+espa%26ntilde%3Boles%3A+un+estudio+transversal&title=Revista+de+Psicolog%C3%ADa+Cl%C3%ADnica+con+Ni%C3%B1os+y+Adolescentes&issn=2340-8340&date=2020-09-01&volume=7&issue=3&spage=27&au=Erades%2C+Nieves%3BMorales%2C+Alexandra&isbn=&jtitle=Revista+de+Psicolog%C3%ADa+Cl%C3%ADnica+con+Ni%C3%B1os+y+Adolescentes&btitle=&rft_id=info:eric/2021-06276-003&rft_id=info:doi/

Esteves, C. S., de Oliveira, C. R., & Argimon, I. I. L. (2020). Social Distancing: Prevalence of Depressive, Anxiety, and Stress Symptoms Among Brazilian Students During the COVID-19 Pandemic [Article]. *Frontiers in public health*, *8*, 589966. <https://doi.org/10.3389/fpubh.2020.589966>

Ezpeleta, L., Navarro, J. B., de la Osa, N., Trepat, E., & Penelo, E. (2020). Life conditions during COVID-19 lockdown and mental health in Spanish adolescents [Article]. *International Journal of Environmental Research and Public Health*, *17*(19), 1-13. <https://doi.org/10.3390/ijerph17197327>

Faigenbaum, A. D., MacDonald, J. P., Carvalho, C., & Rebullido, T. R. (2020). THE PEDIATRIC INACTIVITY TRIAD: A TRIPLE JEOPARDY FOR MODERN DAY YOUTH. *ACSM's Health & Fitness Journal*, *24*(4), 10-17. <https://doi.org/10.1249/FIT.0000000000000584>

Faraji, M., Sepahvand, E., & Rahmati, R. (2021). The effects of covid-19 related quarantine on physical and social pain of children with autism [Article]. *Journal of Pain Management*, *14*(1), 81-84. <https://www.embase.com/search/results?subaction=viewrecord&id=L2013499364&from=export>

Farì, G., Di Paolo, S., Ungaro, D., Luperto, G., Farì, E., & Latino, F. (2021). The Impact of COVID-19 on Sport and Daily Activities in an Italian Cohort of Football School Children. *International Journal of Athletic Therapy & Training*, *26*(5), 274-278. <https://doi.org/10.1123/ijatt.2020-0066>

Feng, Z., Xu, L., Cheng, P., Zhang, L., Li, L. J., & Li, W. H. (2020). The psychological impact of COVID-19 on the families of first-line rescuers [Article]. *Indian Journal of Psychiatry*, *62*(9), S438-S444. <https://doi.org/10.4103/psychiatry.IndianJPsychiatry_1057_20>

Fernández Cruz, M., Álvarez Rodríguez, J., Ávalos Ruiz, I., Cuevas López, M., de Barros Camargo, C., Díaz Rosas, F., González Castellón, E., González González, D., Hernández Fernández, A., Ibáñez Cubillas, P., & Lizarte Simón, E. J. (2020). Evaluation of the Emotional and Cognitive Regulation of Young People in a Lockdown Situation Due to the Covid-19 Pandemic. *Frontiers in Psychology*, *11*, 565503. <https://doi.org/10.3389/fpsyg.2020.565503>

Fernández, R. S., Crivelli, L., Guimet, N. M., Allegri, R. F., & Pedreira, M. E. (2020). Psychological distress associated with COVID-19 quarantine: Latent profile analysis, outcome prediction and mediation analysis. *Journal of Affective Disorders*, *277*, 75-84. <https://doi.org/10.1016/j.jad.2020.07.133>

Ferrante, M. J., Goldsmith, J., Tauriello, S., Epstein, L. H., Leone, L. A., & Anzman-Frasca, S. (2021). Food acquisition and daily life for U.S. families with 4-to 8-year-old children during COVID-19: Findings from a nationally representative survey [Article]. *International Journal of Environmental Research and Public Health*, *18*(4), 1-15. <https://doi.org/10.3390/ijerph18041734>

Foley, S., Badinlou, F., Brocki, K. C., Frick, M. A., Ronchi, L., & Hughes, C. (2021). Family function and child adjustment difficulties in the covid-19 pandemic: An international study [Article]. *International Journal of Environmental Research and Public Health*, *18*(21). <https://doi.org/10.3390/ijerph182111136>

Gabriel, M. G., Brown, A., León, M., & Outley, C. (2020). Power and social control of youth during the covid-19 pandemic. *Leisure Sciences*. <https://doi.org/http://dx.doi.org/10.1080/01490400.2020.1774008>

Ghosh, R., Dubey, M. J., Chatterjee, S., & Dubey, S. (2020). Impact of COVID-19 on children: Special focus on the psychosocial aspect [Review]. *Minerva Pediatrica*, *72*(3), 226-235. <https://doi.org/10.23736/S0026-4946.20.05887-9>

Gjaka, M., Feka, K., Bianco, A., Tishukaj, F., Giustino, V., Parroco, A. M., Palma, A., & Battaglia, G. (2021). The effect of covid-19 lockdown measures on physical activity levels and sedentary behaviour in a relatively young population living in kosovo [Article]. *Journal of Clinical Medicine*, *10*(4), 1-15. <https://doi.org/10.3390/jcm10040763>

Golberstein, E., Wen, H., & Miller, B. F. (2021). Coronavirus Disease 2019 and Effects of School Closure for Children and Their Families-Reply [Letter]. *JAMA Pediatrics*, *175*(2), 211-212. <https://doi.org/10.1001/jamapediatrics.2020.3598>

Gruba, G., Kasiak, P. S., Gębarowska, J., Adamczyk, N., Sikora, Z., Jodczyk, A. M., Mamcarz, A., & Śliż, D. (2021). Pals study of sleep deprivation and mental health consequences of the covid-19 pandemic among university students: A cross-sectional survey [Article]. *International Journal of Environmental Research and Public Health*, *18*(18). <https://doi.org/10.3390/ijerph18189581>

Guan, H., Okely, A. D., Aguilar-Farias, N., del Pozo Cruz, B., Draper, C. E., El Hamdouchi, A., Florindo, A. A., Jáuregui, A., Katzmarzyk, P. T., Kontsevaya, A., Löf, M., Park, W., Reilly, J. J., Sharma, D., Tremblay, M. S., & Veldman, S. L. C. (2020). Promoting healthy movement behaviours among children during the COVID-19 pandemic [Note]. *The Lancet Child and Adolescent Health*, *4*(6), 416-418. <https://doi.org/10.1016/S2352-4642(20)30131-0>

Guo, Y. F., Liao, M. Q., Cai, W. L., Yu, X. X., Li, S. N., Ke, X. Y., Tan, S. X., Luo, Z. Y., Cui, Y. F., Wang, Q., Gao, X. P., Liu, J., Liu, Y. H., Zhu, S., & Zeng, F. F. (2021). Physical activity, screen exposure and sleep among students during the pandemic of COVID-19 [Article]. *Scientific reports*, *11*(1), 8529. <https://doi.org/10.1038/s41598-021-88071-4>

Gupta, S., Schreiber, M., McGuire, T., & Newton, C. (2021). Addressing Pediatric Mental Health during COVID-19 and other Disasters: A National Tabletop Exercise [Article in Press]. *Disaster medicine and public health preparedness*, 1-13. <https://doi.org/10.1017/dmp.2021.122>

Gupta, T., & Nebhinani, N. (2020). Impact of COVID-19 pandemic on child and adolescent mental health [Editorial]. *Journal of Indian Association for Child and Adolescent Mental Health*, *16*(3), 1-16. <https://www.embase.com/search/results?subaction=viewrecord&id=L2004898893&from=export>

Hageman, J. R. (2020). Children’s and families’ behavioral and mental health during COVID-19 [Editorial]. *Pediatric Annals*, *49*(10), e405-e406. <https://doi.org/10.3928/19382359-20200922-05>

Hageman, J. R. (2021). Covid-19 and the mental health effects on adolescents and young adults [Editorial]. *Pediatric Annals*, *50*(8), e308-e309. <https://doi.org/10.3928/19382359-20210721-03>

Hagen, K., Solem, S., Stavrum, A. K., Eid, J., Kvale, G., Samdal, O., & Hellard, S. L. (2021). Mental health symptoms during the first months of the COVID-19 outbreak in Norway: A cross-sectional survey study [Article in Press]. *Scandinavian journal of public health*, 14034948211059525. <https://doi.org/10.1177/14034948211059525>

Hamoda, H. M., Chiumento, A., Alonge, O., Hamdani, S. U., Saeed, K., Wissow, L., & Rahman, A. (2021). Addressing the consequences of the covid-19 lockdown for children's mental health: Investing in school mental health programs [Article]. *Psychiatric Services*, *72*(6), 729-731. <https://doi.org/10.1176/appi.ps.202000597>

Hester, M. (2021). AAP issues interim guidance for nutrition in the pandemic. *Contemporary Pediatrics*, *38*(2), 30-30. <https://search.ebscohost.com/login.aspx?direct=true&AuthType=sso&db=ccm&AN=148660343&site=ehost-live&custid=s3890005>

Hoffman, J. A., & Miller, E. A. (2020). Addressing the Consequences of School Closure Due to COVID-19 on Children's Physical and Mental Well-Being [Note]. *World Medical and Health Policy*, *12*(3), 300-310. <https://doi.org/10.1002/wmh3.365>

Hosen, I., al Mamun, F., & Mamun, M. A. (2021). The role of sociodemographics, behavioral factors, and internet use behaviors in students' psychological health amid COVID-19 pandemic in Bangladesh [Article]. *Health Science Reports*, *4*(4). <https://doi.org/10.1002/hsr2.398>

Idoiaga, N., Berasategi, N., Eiguren, A., & Picaza, M. (2020). Exploring Children's Social and Emotional Representations of the COVID-19 Pandemic. *Frontiers in Psychology*, *11*, 1952. <https://doi.org/10.3389/fpsyg.2020.01952>

Ijaz, S. F., & Ijaz, I. (2021). Impact of excessive screen use on sleep habits in children during covid-19 pandemic [Article]. *Pakistan Paediatric Journal*, *45*(4), 395-400. <https://www.embase.com/search/results?subaction=viewrecord&id=L2015114839&from=export>

The Impact of COVID-19 on the Well-Being of Division III Student-Athletes. (2020). *Sport Journal*, N.PAG-N.PAG. <https://search.ebscohost.com/login.aspx?direct=true&AuthType=sso&db=ccm&AN=146379233&site=ehost-live&custid=s3890005>

The Impact of COVID-19 Pandemic on High Performance Secondary School Student-Athletes. (2020). *Sport Journal*, N.PAG-N.PAG. <https://search.ebscohost.com/login.aspx?direct=true&AuthType=sso&db=ccm&AN=144766703&site=ehost-live&custid=s3890005>

Iozzi, L., Brambilla, I., Foiadelli, T., Marseglia, G. L., & Ciprandi, G. (2020). Paediatric emergency department visits fell by more than 70% during the COVID-19 lockdown in Northern Italy. *Acta Paediatrica*, *109*(10), 2137-2138. <https://doi.org/http://dx.doi.org/10.1111/apa.15458>

Iqbal, S. A., & Tayyab, N. (2021). COVID-19 and children: The mental and physical reverberations of the pandemic [Letter]. *Child: Care, Health and Development*, *47*(1), 136-139. <https://doi.org/10.1111/cch.12822>

Jarnig, G., Jaunig, J., & van Poppel, M. N. M. (2021). Association of COVID-19 Mitigation Measures With Changes in Cardiorespiratory Fitness and Body Mass Index Among Children Aged 7 to 10 Years in Austria [Journal Article; Randomized Controlled Trial; Research Support, Non‐U.S. Gov't]. *JAMA Network Open*, *4*(8), e2121675. <https://doi.org/10.1001/jamanetworkopen.2021.21675>

Jolliff, A., Zhao, Q., Eickhoff, J., & Moreno, M. (2021). Depression, Anxiety, and Daily Activity Among Adolescents Before and During the COVID-19 Pandemic: Cross-sectional Survey Study. *JMIR formative research*, *5*(12), e30702. <https://doi.org/10.2196/30702>

José Puccinelli, P., Santos da Costa, T., Seffrin, A., Barbosa de Lira, C. A., Luiz Vancini, R., Nikolaidis, P. T., Knechtle, B., Rosemann, T., Hill, L., & Santos Andrade, M. (2021). Reduced level of physical activity during COVID-19 pandemic is associated with depression and anxiety levels: an internet-based survey. *BMC Public Health*, *21*(1), 1-11. <https://doi.org/10.1186/s12889-021-10470-z>

Jurak, G., Morrison, S. A., Kovač, M., Leskošek, B., Sember, V., Strel, J., & Starc, G. (2021). A COVID-19 Crisis in Child Physical Fitness: Creating a Barometric Tool of Public Health Engagement for the Republic of Slovenia [Article]. *Frontiers in public health*, *9*, 644235. <https://doi.org/10.3389/fpubh.2021.644235>

Kalvin, C. B., Jordan, R. P., Rowley, S. N., Weis, A., Wood, K. S., Wood, J. J., Ibrahim, K., & Sukhodolsky, D. G. (2021). Conducting CBT for Anxiety in Children with Autism Spectrum Disorder During COVID-19 Pandemic [Note]. *Journal of Autism and Developmental Disorders*, *51*(11), 4239-4247. <https://doi.org/10.1007/s10803-020-04845-1>

Kerekes, N., Bador, K., Sfendla, A., Belaatar, M., El Mzadi, A., Jovic, V., Damjanovic, R., Erlandsson, M., Nguyen, H. T. M., Nguyen, N. T. A., Ulberg, S. F., Kuch-Cecconi, R. H., Meszaros, Z. S., Stevanovic, D., Senhaji, M., Ahlström, B. H., & Zouini, B. (2021). Changes in adolescents’ psychosocial functioning and well-being as a consequence of long-term covid-19 restrictions [Article]. *International Journal of Environmental Research and Public Health*, *18*(16). <https://doi.org/10.3390/ijerph18168755>

Khan, A. S. (2020). Impact of COVID-19 pandemic and subsequent lockdown on quality of life of high-school students [Article]. *Pakistan Journal of Medical and Health Sciences*, *14*(4), 997-999. <https://www.embase.com/search/results?subaction=viewrecord&id=L2010623619&from=export>

Kim, S. J., Lee, S., Han, H., Jung, J., Yang, S. J., & Shin, Y. (2021). Parental Mental Health and Children's Behaviors and Media Usage during COVID-19-Related School Closures [Article]. *Journal of Korean medical science*, *36*(25), e184. <https://doi.org/10.3346/jkms.2021.36.e184>

Kim, S. Y., Yoo, D. M., Min, C., & Choi, H. G. (2021). Changes in Dietary Habits and Exercise Pattern of Korean Adolescents from Prior to during the COVID-19 Pandemic. *Nutrients*, *13*(10). <https://doi.org/10.3390/nu13103314>

Kiss, O., Alzueta, E., Yuksel, D., Pohl, K. M., de Zambotti, M., Műller-Oehring, E. M., Prouty, D., Durley, I., Pelham, W. E., McCabe, C. J., Gonzalez, M. R., Brown, S. A., Wade, N. E., Marshall, A. T., Sowell, E. R., Breslin, F. J., Lisdahl, K. M., Dick, A. S., Sheth, C. S., . . . Baker, F. C. (2022). The Pandemic's Toll on Young Adolescents: Prevention and Intervention Targets to Preserve Their Mental Health [Article]. *Journal of Adolescent Health*, *70*(3), 387-395. <https://doi.org/10.1016/j.jadohealth.2021.11.023>

Kuygun Karci, C., & Arici Gurbuz, A. (2021). Challenges of children and adolescents with attention-deficit/hyperactivity disorder during the covid-19 pandemic. *Nordic Journal of Psychiatry*. <https://doi.org/http://dx.doi.org/10.1080/08039488.2021.1980610>

Lange, S. J., Kompaniyets, L., Freedman, D. S., Kraus, E. M., Porter, R., Blanck, H. M., & Goodman, A. B. (2021). Longitudinal Trends in Body Mass Index Before and During the COVID-19 Pandemic Among Persons Aged 2-19 Years - United States, 2018-2020 [Article]. *MMWR. Morbidity and mortality weekly report*, *70*(37), 1278-1283. <https://doi.org/10.15585/mmwr.mm7037a3>

Lanza, K., Durand, C. P., Alcazar, M., Ehlers, S., Zhang, K., & Kohl, H. W. (2021). School parks as a community health resource: use of joint-use parks by children before and during covid-19 pandemic [Article]. *International Journal of Environmental Research and Public Health*, *18*(17). <https://doi.org/10.3390/ijerph18179237>

Lee, S.-M., So, W.-Y., & Youn, H.-S. (2021). Importance-Performance Analysis of Health Perception among Korean Adolescents during the COVID-19 Pandemic. *International Journal of Environmental Research and Public Health*, *18*(3). <https://doi.org/10.3390/ijerph18031280>

Lessard, L. M., & Puhl, R. M. (2021). Adolescents' Exposure to and Experiences of Weight Stigma During the COVID-19 Pandemic [Article]. *Journal of Pediatric Psychology*, *46*(8), 950-959. <https://doi.org/10.1093/jpepsy/jsab071>

Leung, K. K. Y., Chu, S. P. W., Hon, K. L., & Leung, T. F. (2021). Indirect consequences of covid-19 on children’s health [Letter]. *Hong Kong Medical Journal*, *27*(2), 160. <https://doi.org/10.12809/hkmj208694>

Li, S. H., Beames, J. R., Newby, J. M., Maston, K., Christensen, H., & Werner-Seidler, A. (2021). The impact of COVID-19 on the lives and mental health of Australian adolescents [Article in Press]. *European Child and Adolescent Psychiatry*. <https://doi.org/10.1007/s00787-021-01790-x>

Li, W., Wang, Z., Wang, G., Ip, P., Sun, X., Jiang, Y., & Jiang, F. (2021). Socioeconomic inequality in child mental health during the COVID-19 pandemic: First evidence from China [Article]. *Journal of Affective Disorders*, *287*, 8-14. <https://doi.org/10.1016/j.jad.2021.03.009>

Li, Y., Zhao, J., Ma, Z., McReynolds, L. S., Lin, D., Chen, Z., Wang, T., Wang, D., Zhang, Y., Zhang, J., Fan, F., & Liu, X. (2021). Mental Health Among College Students During the COVID-19 Pandemic in China: A 2-Wave Longitudinal Survey [Article]. *Journal of Affective Disorders*, *281*, 597-604. <https://doi.org/10.1016/j.jad.2020.11.109>

Li, Z. L., Liu, R., He, F., Li, S. Y., Zhao, Y. J., Zhang, W. Y., Zhang, Y., Cheung, T., Jackson, T., Tang, Y. L., & Xiang, Y. T. (2021). Prevalence of Internet Addiction Disorder and Its Correlates Among Clinically Stable Adolescents With Psychiatric Disorders in China During the COVID-19 Outbreak [Article]. *Frontiers in Psychiatry*, *12*. <https://doi.org/10.3389/fpsyt.2021.686177>

Liao, S., Luo, B., Liu, H., Zhao, L., Shi, W., Lei, Y., & Jia, P. (2021). Bilateral associations between sleep duration and depressive symptoms among Chinese adolescents before and during the COVID-19 pandemic [Article]. *Sleep Medicine*, *84*, 289-293. <https://doi.org/10.1016/j.sleep.2021.06.007>

Lin, J., Guo, T., Becker, B., Yu, Q., Chen, S.-T., Brendon, S., Hossain, M. M., Cunha, P. M., Soares, F. C., Veronese, N., Yu, J. J., Grabovac, I., Smith, L., Yeung, A., Zou, L., & Li, H. (2020). Depression is Associated with Moderate-Intensity Physical Activity Among College Students During the COVID-19 Pandemic: Differs by Activity Level, Gender and Gender Role. *Psychology research and behavior management*, *13*, 1123-1134. <https://doi.org/10.2147/PRBM.S277435>

Linck, D. T. (2018). Female Teens Step It Up with the Fitbit Zip: A Randomized Controlled Pilot Study. *Female Teens Step It Up With The Fitbit Zip: A Randomized Controlled Pilot Study*, 1-1. <https://search.ebscohost.com/login.aspx?direct=true&AuthType=sso&db=ccm&AN=131799034&site=ehost-live&custid=s3890005>

Lindoso, L., Astley, C., Queiroz, L. B., Gualano, B., Pereira, R. M. R., Tannuri, U., Campos, L. M. M. D. A., Lourenço, B., Toma, R. K., Medeiros, K., Watanabe, A., Moreno Grangeiro, P., Barros, V. D. P. M. F. R., Casella, C. B., Farhat, S., Polanczyk, G. V., & Silva, C. A. (2021). Physical and mental health impacts during COVID-19 quarantine in adolescents with preexisting chronic immunocompromised conditions [Article in Press]. *Jornal de Pediatria*. <https://doi.org/10.1016/j.jped.2021.09.002>

Lindsay, R., Trott, M., Allen, P., & Smith, L. (2021). Covid-19 and children’s mental health [Editorial]. *Jornal Brasileiro de Psiquiatria*, *70*(2), 87-88. <https://doi.org/10.1590/0047-2085000000330>

Logan, L. M., Ciftci, B., Longoni, G., Berenbaum, T., Ly, M., Stephens, S., & Yeh, A. (2021). Effects of COVID-19 on Mental Health, Physical Activity and Sleep in Pediatric Neuroinflammatory Disorders [Conference Abstract]. *Multiple Sclerosis Journal*, *27*(1 SUPPL), 117-118. <https://doi.org/10.1177/13524585211015908>

Logan, L. M., Kavaklioglu, B. C., Longoni, G., Berenbaum, T., Ly, M., Stephens, S., & Yeh, E. A. (2021). Impact of the COVID-19 pandemic on mental health, physical activity and sleep in children with neuroinflammatory disorders [Conference Abstract]. *Neurology*, *96*(15 SUPPL 1). <https://www.embase.com/search/results?subaction=viewrecord&id=L635945859&from=export>

López-Aymes, G., Valadez, M. L. D., Rodríguez-Naveiras, E., Castellanos-Simons, D., Aguirre, T., & Borges, Á. (2021). A Mixed Methods Research Study of Parental Perception of Physical Activity and Quality of Life of Children Under Home Lock Down in the COVID-19 Pandemic. *Front Psychol*, *12*, 649481. <https://doi.org/10.3389/fpsyg.2021.649481>

López-Bueno, R., López-Sánchez, G. F., Casajús, J. A., Calatayud, J., Gil-Salmerón, A., Grabovac, I., Tully, M. A., & Smith, L. (2020). Health-Related Behaviors Among School-Aged Children and Adolescents During the Spanish Covid-19 Confinement [Article]. *Frontiers in Pediatrics*, *8*. <https://doi.org/10.3389/fped.2020.00573>

López-Gil, J. F., R. Gaya, A., Reuter, C. P., Caetano, C. I., Gomes Sentone, R., Silva Caetano, H. B., & Brazo-Sayavera, J. (2021). Sleep-related problems and eating habits during COVID-19 lockdown in a southern Brazilian youth sample [Article]. *Sleep Medicine*, *85*, 150-156. <https://doi.org/10.1016/j.sleep.2021.07.003>

López-Gil, J. F., Tremblay, M. S., & Brazo-Sayavera, J. (2021). Changes in Healthy Behaviors and Meeting 24-h Movement Guidelines in Spanish and Brazilian Preschoolers, Children and Adolescents during the COVID-19 Lockdown. *Children*, *8*(2), 1-10. <https://doi.org/10.3390/children8020083>

Lorch, M., & Fuchs, D. (2020). COVID-19: Effects of the shutdown on children and families in child and youth care services in Germany. *Child & Youth Services*, *41*(3), 290-292. <https://doi.org/http://dx.doi.org/10.1080/0145935X.2020.1835166>

Lourenço, A., Martins, F., Pereira, B., & Mendes, R. (2021). Children are back to school, but is play still in lockdown? Play experiences, social interactions, and children’s quality of life in primary education in the covid-19 pandemic in 2020 [Article]. *International Journal of Environmental Research and Public Health*, *18*(23). <https://doi.org/10.3390/ijerph182312454>

Luijten, M., Muilekom, M. V., Teela, L., Oers, H. V., Oostrom, K., & Haverman, L. (2019). How the COVID-19 pandemic impacts the psychosocial well-being of children and adolescents in the Netherlands [Conference Abstract]. *Quality of Life Research*, *29*(SUPPL 1), S64. <https://doi.org/10.1007/s11136-020-02626-y>

Luo, L., Song, N., Yang, H., Huang, J., Zhou, L., & Zhang, L. (2021). Intervention Effect of Long-Term Aerobic Training on Anxiety, Depression, and Sleep Quality of Middle School Students With Depression After COVID-19 [Article]. *Frontiers in Psychiatry*, *12*. <https://doi.org/10.3389/fpsyt.2021.720833>

MacKenzie, N. E., Keys, E., Hall, W. A., Gruber, R., Smith, I. M., Constantin, E., Godbout, R., Stremler, R., Reid, G. J., Hanlon-Dearman, A., Brown, C. A., Shea, S., Weiss, S. K., Ipsiroglu, O., Witmans, M., Chambers, C. T., Andreou, P., Begum, E., & Corkum, P. (2021). Children's Sleep During COVID-19: How Sleep Influences Surviving and Thriving in Families [Article]. *Journal of Pediatric Psychology*, *46*(9), 1051-1062. <https://doi.org/10.1093/jpepsy/jsab075>

Margaritis, I., Houdart, S., El Ouadrhiri, Y., Bigard, X., Vuillemin, A., & Duché, P. (2020). How to deal with COVID-19 epidemic-related lockdown physical inactivity and sedentary increase in youth? Adaptation of Anses' benchmarks. *Archives of Public Health*, *78*(1), 1-6. <https://doi.org/10.1186/s13690-020-00432-z>

Martinez, S. M., Tschann, J. M., Butte, N. F., & Grandner, M. (2019). Sleep longer, be active, and eat healthily: 24-hour circadian-related behaviors are protective of children's weight status [Conference Abstract]. *Sleep*, *42*, A321. <https://doi.org/10.1093/sleep/zsz067.798>

Mc Guine, T., Biese, K., Hetzel, S., Kliethermes, S., Reardon, C., Bell, D., Brooks, M. A., & Watson, A. (2021). The impact of covid-19 related school closures and sport cancellations on the health of adolescent athletes [Conference Abstract]. *Orthopaedic Journal of Sports Medicine*, *9*(7 SUPPL 3). <https://doi.org/10.1177/2325967121S000170>

McCluskey, G., Fry, D., Hamilton, S., King, A., Laurie, M., McAra, L., & Stewart, T. M. (2021). School closures, exam cancellations and isolation: the impact of Covid-19 on young people’s mental health [Article]. *Emotional and Behavioural Difficulties*, *26*(1), 46-59. <https://doi.org/10.1080/13632752.2021.1903182>

McCormack, G. R., Doyle-Baker, P. K., Petersen, J. A., & Ghoneim, D. (2020). Parent anxiety and perceptions of their child's physical activity and sedentary behaviour during the COVID-19 pandemic in Canada [Article]. *Preventive Medicine Reports*, *20*. <https://doi.org/10.1016/j.pmedr.2020.101275>

McDonnell, T., Barrett, M., McNicholas, F., Barrett, E., Conlon, C., Cummins, F., Hensey, C., McAuliffe, E., & Nicholson, E. (2021). Increased mental health presentations by children aged 5-15 at emergency departments during the first 12 months of covid-19 [Article]. *Irish Medical Journal*, *114*(5). <https://www.embase.com/search/results?subaction=viewrecord&id=L2007872337&from=export>

McGuine, T., Biese, K., Hetzel, S., Kliethermes, S., Reardon, C., Bell, D., Brooks, M. A., & Watson, A. (2021). THE IMPACT OF COVID-19 RELATED SCHOOL CLOSURES AND SPORT CANCELLATIONS ON THE HEALTH OF ADOLESCENT ATHLETES...Pediatric Research in Sports Medicine (PRiSM), 8th Annual Meeting, 28-30 January, 2021. *Orthopaedic Journal of Sports Medicine*, *19*, 306-306. <https://doi.org/10.1177/2325967121S00170>

McGuine, T. A., Biese, K., Hetzel, S. J., Schwarz, A., Kliethermes, S., Reardon, C. L., Bell, D. R., Brooks, M. A., & Watson Md, A. M. (2021). High School Sports During the CoVID-19 Pandemic: The Impact of Sport Participation on the Health of Adolescents [Article in Press]. *Journal of athletic training*. <https://doi.org/10.4085/1062-6050-0121.21>

McGuine, T. A., Biese, K. M., Petrovska, L., Hetzel, S. J., Reardon, C., Kliethermes, S., Bell, D. R., Brooks, A., & Watson, A. M. (2020). The Health Of Us Adolescent Athletes During Covid-19 Related School Closures And Sport Cancellations [Article in Press]. *Journal of athletic training*. <https://doi.org/10.4085/478-20>

McGuine, T. A., Biese, K. M., Petrovska, L., Hetzel, S. J., Reardon, C. L., Kliethermes, S., Bell, D. R., Brooks, A., & Watson, A. M. (2021). Changes in the Health of Adolescent Athletes: A Comparison of Health Measures Collected Before and During the COVID-19 Pandemic [Article]. *Journal of athletic training*, *56*(8), 836-844. <https://doi.org/10.4085/1062-6050-0739.20>

Mehdizadeh, A., Shafiee, M., Khadem-Rezaiyan, M., Sardar, M. A., Vatanparast, H., Rose, E., Rajabzadeh, M., & Nematy, M. (2019). Evidence for the Validity of the Children's Attraction to Physical Activity (CAPA) Scale in Iranian Preschool Children [Article]. *Journal of Pediatric Nursing*, *44*, e52-e57. <https://doi.org/10.1016/j.pedn.2018.10.021>

Milajerdi, H. R., Amirshaghaghi, F., Milajerdi, S. R., Mojavery, L. E., Panahibakhsh, M., & Najafabadi, M. G. (2021). The effect of sedentary behavior on physical and social pain of children during COVID-19 quarantine: Motor and mental recommendation [Article]. *Journal of Pain Management*, *14*(2), 155-158. <https://www.embase.com/search/results?subaction=viewrecord&id=L2014710152&from=export>

Miller, R. L., Moran, M., Shomaker, L. B., Seiter, N., Sanchez, N., Verros, M., Rayburn, S., Johnson, S., & Lucas-Thompson, R. (2021). Health effects of COVID-19 for vulnerable adolescents in a randomized controlled trial. *School Psychology*, *36*(5), 293-302. <https://doi.org/http://dx.doi.org/10.1037/spq0000458>

Mingazova, E. N., Akimova, L. V., Pozharskaya, E. N., & Mingazov, R. N. (2021). Health risks of school-age children with distance learning in the first months of the spread of COVID-19 [Article]. *Problemy sotsial'noi gigieny, zdravookhraneniia i istorii meditsiny*, *29*(Issue), 588-592. <https://doi.org/10.32687/0869-866X-2021-29-s1-588-592>

Mitra, R., Moore, S. A., Gillespie, M., Faulkner, G., Vanderloo, L. M., Chulak-Bozzer, T., Rhodes, R. E., Brussoni, M., & Tremblay, M. S. (2020). Healthy movement behaviours in children and youth during the COVID-19 pandemic: Exploring the role of the neighbourhood environment. *Health & Place*, *65*, 9. <https://doi.org/http://dx.doi.org/10.1016/j.healthplace.2020.102418>

Mittal, V. A., Firth, J., & Kimhy, D. (2020). Combating the Dangers of Sedentary Activity on Child and Adolescent Mental Health During the Time of COVID-19 [Letter]. *Journal of the American Academy of Child and Adolescent Psychiatry*, *59*(11), 1197-1198. <https://doi.org/10.1016/j.jaac.2020.08.003>

Mohammadzadeh, F., Delshad Noghabi, A., Khosravan, S., Bazeli, J., Armanmehr, V., & Paykani, T. (2020). Anxiety Severity Levels and Coping Strategies during the COVID-19 Pandemic among People Aged 15 Years and Above in Gonabad, Iran. *Archives of Iranian Medicine*, *23*(9), 633-638. <https://doi.org/10.34172/aim.2020.76>

Moore, H. J., Nixon, C. A., Lake, A. A., Douthwaite, W., O'Malley, C. L., Pedley, C. L., Summerbell, C. D., & Routen, A. C. (2014). The environment can explain differences in adolescents’ daily physical activity levels living in a deprived urban area: Cross-sectional study using accelerometry, GPS, and focus groups. *Journal of Physical Activity & Health*, *11*(8), 1517-1524. <https://doi.org/http://dx.doi.org/10.1123/jpah.2012-0420>

Moore, S. A., Sharma, R., Martin Ginis, K. A., & Arbour-Nicitopoulos, K. P. (2021). Parental Support Is Associated With Healthy Movement Behaviours In Children With Disabilities During Covid-19. *Medicine & Science in Sports & Exercise*, *53*, 222-222. <https://doi.org/10.1249/01.mss.0000761632.15601.ba>

Morales, J., Fukuda, D. H., Garcia, V., Pierantozzi, E., Curto, C., Martínez-Ferrer, J. O., Gómez, A. M., Carballeira, E., & Guerra-Balic, M. (2021). Behavioural improvements in children with autism spectrum disorder after participation in an adapted judo programme followed by deleterious effects during the COVID-19 lockdown [Article]. *International Journal of Environmental Research and Public Health*, *18*(16). <https://doi.org/10.3390/ijerph18168515>

Morgül, E., Kallitsoglou, A., & Essau, C. A. (2020). Psychological effects of the COVID-19 lockdown on children and families in the UK. *Revista de Psicología Clínica con Niños y Adolescentes*, *7*(3), 42-48. <https://www.proquest.com/scholarly-journals/psychological-effects-covid-19-lockdown-on/docview/2501283083/se-2?accountid=164166>

Morres, I. D., Galanis, E., Hatzigeorgiadis, A., Androutsos, O., & Theodorakis, Y. (2021). Physical activity, sedentariness, eating behaviour and well-being during a covid-19 lockdown period in greek adolescents [Article]. *Nutrients*, *13*(5). <https://doi.org/10.3390/nu13051449>

Munasinghe, S., Sperandei, S., Freebairn, L., Conroy, E., Jani, H., Marjanovic, S., & Page, A. (2020). The Impact of Physical Distancing Policies During the COVID-19 Pandemic on Health and Well-Being Among Australian Adolescents [Article]. *Journal of Adolescent Health*, *67*(5), 653-661. <https://doi.org/10.1016/j.jadohealth.2020.08.008>

Muñoz-Fernández, N., & Rodríguez-Meirinhos, A. (2021). Adolescents’ concerns, routines, peer activities, frustration, and optimism in the time of covid-19 confinement in spain [Article]. *Journal of Clinical Medicine*, *10*(4), 1-13. <https://doi.org/10.3390/jcm10040798>

Muratori, P., & Ciacchini, R. (2020). Children and the COVID-19 transition: Psychological reflections and suggestions on adapting to the emergency [Article]. *Clinical Neuropsychiatry*, *17*(2), 131-134. <https://doi.org/10.36131/CN20200219>

Nakajima, R., Kamada, H., Kasai, T., Tomaru, Y., Waku, M., Yamaki, A., Ban, A., Miyakawa, S., Yamazaki, M., & Shiraki, H. (2021). Effect of temporary school closure due to COVID-19 on musculoskeletal function in elementary school children. *Journal of rural medicine : JRM*, *16*(3), 154-159. <https://doi.org/10.2185/jrm.2021-006>

Nct. (2021). Physical Training and Diet for Childhood Obesity. [*https://clinicaltrials.gov/show/NCT04789525*](https://clinicaltrials.gov/show/NCT04789525). <https://www.cochranelibrary.com/central/doi/10.1002/central/CN-02249746/full>

Neshteruk, C. D., Zizzi, A., Suarez, L., Erickson, E., Kraus, W. E., Li, J. S., Skinner, A. C., Story, M., Zucker, N., & Armstrong, S. C. (2021). Weight-Related Behaviors of Children with Obesity during the COVID-19 Pandemic [Journal Article; Randomized Controlled Trial; Research Support, Non‐U.S. Gov't]. *Childhood obesity (Print)*, *17*(6), 371‐378. <https://doi.org/10.1089/chi.2021.0038>

Ng, K., Cooper, J., McHale, F., Clifford, J., & Woods, C. (2020). Barriers and facilitators to changes in adolescent physical activity during COVID-19. *BMJ open sport & exercise medicine*, *6*(1), e000919. <https://doi.org/10.1136/bmjsem-2020-000919>

Ng, K., Cosma, A., Svacina, K., Boniel-Nissim, M., & Badura, P. (2021). Czech adolescents’ remote school and health experiences during the spring 2020 COVID-19 lockdown [Article]. *Preventive Medicine Reports*, *22*. <https://doi.org/10.1016/j.pmedr.2021.101386>

Nie, Y., Ma, Y., Li, X., Wu, Y., Liu, W., Tan, Z., Li, J., Zhang, C., Lv, C., & Liu, T. (2021). PA during the COVID-19 outbreak in China: a cross-sectional study. *Neural computing & applications*, 1-16. <https://doi.org/10.1007/s00521-021-06538-x>

Nobari, H., Fashi, M., Eskandari, A., Villafaina, S., Murillo-Garcia, Á., & Pérez-Gómez, J. (2021). Effect of covid-19 on health-related quality of life in adolescents and children: A systematic review [Review]. *International Journal of Environmental Research and Public Health*, *18*(9). <https://doi.org/10.3390/ijerph18094563>

Nyberg, G. (2021). It is time to get a move on and tackle worrying health behaviour patterns in children and adolescents. *Acta Paediatrica*, *110*(9), 2499-2500. <https://doi.org/10.1111/apa.15891>

O'Donoghue, B., Castagnini, E., Langstone, A., Mifsud, N., Thompson, A., Killackey, E., & McGorry, P. (2021). Sedentary behaviour in young people presenting with a first episode of psychosis before and during the covid-19 pandemic restrictions [Letter]. *Schizophrenia Research*, *233*, 31-33. <https://doi.org/10.1016/j.schres.2021.06.006>

O'Kane, S. M., Lahart, I. M., Gallagher, A. M., Carlin, A., Faulkner, M., Jago, R., & Murphy, M. H. (2021). Changes in Physical Activity, Sleep, Mental Health, and Social Media Use During COVID-19 Lockdown Among Adolescent Girls: A Mixed-Methods Study [Article]. *Journal of Physical Activity & Health*, *18*(6), 677-685. <https://doi.org/10.1123/jpah.2020-0649>

O'Loughlin, S., Sharaf, S., & Van Der Spek, N. (2021). Changing patterns in paediatric attendances during the covid-19 pandemic [Conference Abstract]. *Archives of Disease in Childhood*, *106*(SUPPL 1), A345. <https://doi.org/10.1136/archdischild-2021-rcpch.600>

O’rourke, T., Dale, R., Humer, E., Probst, T., Plener, P., & Pieh, C. (2022). Health Behaviors in Austrian Apprentices and School Students during the COVID-19 Pandemic [Article]. *International Journal of Environmental Research and Public Health*, *19*(3). <https://doi.org/10.3390/ijerph19031049>

Okely, A. D., Kariippanon, K. E., Guan, H., Taylor, E. K., Suesse, T., Cross, P. L., Chong, K. H., Suherman, A., Turab, A., Staiano, A. E., Ha, A. S., El Hamdouchi, A., Baig, A., Poh, B. K., Del Pozo-Cruz, B., Chan, C. H. S., Nyström, C. D., Koh, D., Webster, E. K., . . . Draper, C. E. (2021). Global effect of COVID-19 pandemic on physical activity, sedentary behaviour and sleep among 3- to 5-year-old children: a longitudinal study of 14 countries [Article]. *BMC Public Health*, *21*(1), 940. <https://doi.org/10.1186/s12889-021-10852-3>

Okuyama, J., Seto, S., Fukuda, Y., Funakoshi, S., Amae, S., Onobe, J., Izumi, S., Ito, K., & Imamura, F. (2021). Mental health and physical activity among children and adolescents during the COVID-19 pandemic [Article]. *Tohoku Journal of Experimental Medicine*, *253*(3), 203-215. <https://doi.org/10.1620/tjem.253.203>

Oliva, S., Russo, G., Gili, R., Russo, L., Di Mauro, A., Spagnoli, A., Alunni Fegatelli, D., Romani, M., Costa, A., Veraldi, S., & Manti, F. (2021). Risks and Protective Factors Associated With Mental Health Symptoms During COVID-19 Home Confinement in Italian Children and Adolescents: The #Understandingkids Study [Article]. *Frontiers in Pediatrics*, *9*. <https://doi.org/10.3389/fped.2021.664702>

Owen, A., & Bould, K. (2021). Reduced physical activity and increased sedentary behaviour: the damage on young people during the COVID-19 pandemic. *British Journal of Child Health*, *2*(2), 64-68. <https://doi.org/10.12968/chhe.2021.2.2.64>

Pagoto, S. L., & Conroy, D. E. (2021). Revitalizing Adolescent Health Behavior after the COVID-19 Pandemic [Editorial]. *JAMA Pediatrics*, *175*(7), 677-679. <https://doi.org/10.1001/jamapediatrics.2021.0547>

Paterson, D. C., Ramage, K., Moore, S. A., Riazi, N., Tremblay, M. S., & Faulkner, G. (2021). Exploring the impact of COVID-19 on the movement behaviors of children and youth: A scoping review of evidence after the first year [Review]. *Journal of Sport and Health Science*, *10*(6), 675-689. <https://doi.org/10.1016/j.jshs.2021.07.001>

Patra, S., & Patro, B. K. (2021). COVID-19, lockdowns and internet access: Is it pushing adolescents towards suicide? [Article]. *Journal of Indian Association for Child and Adolescent Mental Health*, *17*(2), 216-218. <https://www.embase.com/search/results?subaction=viewrecord&id=L2006944048&from=export>

Patterson, R. R., Sornalingam, S., & Cooper, M. (2021). Consequences of covid-19 on the childhood obesity epidemic [Letter]. *The BMJ*, *373*. <https://doi.org/10.1136/bmj.n953>

Peralta, G. P., Camerini, A. L., Haile, S. R., Kahlert, C. R., Lorthe, E., Marciano, L., Nussbaumer, A., Radtke, T., Ulyte, A., Puhan, M. A., & Kriemler, S. (2021). Lifestyle behaviours of children and adolescents during the first two waves of the COVID-19 pandemic in Switzerland and their relation to well-being: a population-based study. In.

Phd, A. L. (2021). 78. The Effects of the COVID-19 Pandemic on Physical Activity and Mental Well-Being in Older Adolescents in College [Conference Abstract]. *Journal of Adolescent Health*, *68*(2), S42. <https://doi.org/10.1016/j.jadohealth.2020.12.087>

Pierce, E., Ratnaseelan, A., McCracken, E., Langdon, R., Strelzik, J., Fletcher, A., Borner, K., & DiSabella, M. (2021). Pediatric Headache Experience during the COVID-19 Pandemic [Conference Abstract]. *Annals of Neurology*, *90*(SUPPL 26), S46. <https://doi.org/10.1002/ana.26177>

Pietrabissa, G., Volpi, C., Bottacchi, M., Bertuzzi, V., Guerrini Usubini, A., Löffler-Stastka, H., Prevendar, T., Rapelli, G., Cattivelli, R., Castelnuovo, G., Molinari, E., & Sartorio, A. (2021). The impact of social isolation during the covid-19 pandemic on physical and mental health: The lived experience of adolescents with obesity and their caregivers [Article]. *International Journal of Environmental Research and Public Health*, *18*(6), 1-20. <https://doi.org/10.3390/ijerph18063026>

Pietrobelli, A., Pecoraro, L., Ferruzzi, A., Heo, M., Faith, M., Zoller, T., Antoniazzi, F., Piacentini, G., Fearnbach, S. N., & Heymsfield, S. B. (2020). Effects of COVID-19 Lockdown on Lifestyle Behaviors in Children with Obesity Living in Verona, Italy: A Longitudinal Study [Article]. *Obesity*, *28*(8), 1382-1385. <https://doi.org/10.1002/oby.22861>

Pigaiani, Y., Zoccante, L., Zocca, A., Arzenton, A., Menegolli, M., Fadel, S., Ruggeri, M., & Colizzi, M. (2020). Adolescent Lifestyle Behaviors, Coping Strategies and Subjective Wellbeing during the COVID-19 Pandemic: An Online Student Survey. *Healthcare (Basel, Switzerland)*, *8*(4). <https://doi.org/10.3390/healthcare8040472>

Pigeaud, L., de Veld, L., van Hoof, J., & van der Lely, N. (2021). Acute Alcohol Intoxication in Dutch Adolescents Before, During, and After the First COVID-19 Lockdown. *J Adolesc Health*, *69*(6), 905-909. <https://doi.org/10.1016/j.jadohealth.2021.07.038>

Piña, A., Mirhajianmoghadam, H., & Ostrin, L. A. (2021). Objective and Subjective Behavioral Measures in Myopic and Non-Myopic Children during the COVID-19 Pandemic. *Investigative ophthalmology & visual science*, *62*(8), 1986-1986.

Pombo, A., Luz, C., Rodrigues, L. P., & Cordovil, R. (2021). Effects of COVID-19 Confinement on the Household Routines Of Children in Portugal. *Journal of Child & Family Studies*, *30*(7), 1664-1674. <https://doi.org/10.1007/s10826-021-01961-z>

Pombo, A., Luz, C., Rodrigues, L. P., Ferreira, C., & Cordovil, R. (2020). Correlates of children's physical activity during the COVID-19 confinement in Portugal. *Public Health*, *189*, 14-19. <https://doi.org/10.1016/j.puhe.2020.09.009>

Przybylski, R., Craig, M., Lippmann, M., Mah, D. Y., Shafer, K. M., Gauthier, N. S., de Ferranti, S. D., Triedman, J. K., & Alexander, M. E. (2021). Activity During the COVID-19 Pandemic in Children with Cardiac Rhythm Management Devices [Article in Press]. *Pediatric Cardiology*. <https://doi.org/10.1007/s00246-021-02787-8>

Qi, H., Liu, R., Chen, X., Yuan, X. F., Li, Y. Q., Huang, H. H., Zheng, Y., & Wang, G. (2020). Prevalence of anxiety and associated factors for Chinese adolescents during the COVID-19 outbreak [Letter]. *Psychiatry and Clinical Neurosciences*, *74*(10), 555-557. <https://doi.org/10.1111/pcn.13102>

Qin, J., Ding, Y., Gao, J., Wu, Y., Lv, H., & Wu, J. (2021). Effects of COVID-19 on Mental Health and Anxiety of Adolescents Aged 13–16 Years: A Comparative Analysis of Longitudinal Data From China [Article]. *Frontiers in Psychiatry*, *12*. <https://doi.org/10.3389/fpsyt.2021.695556>

Qin, Z., Shi, L., Xue, Y., Lin, H., Zhang, J., Liang, P., Lu, Z., Wu, M., Chen, Y., Zheng, X., Qian, Y., Ouyang, P., Zhang, R., Yi, X., & Zhang, C. (2021). Prevalence and Risk Factors Associated With Self-reported Psychological Distress Among Children and Adolescents During the COVID-19 Pandemic in China. *JAMA Network Open*, *4*(1), e2035487. <https://doi.org/10.1001/jamanetworkopen.2020.35487>

Racine, N., Cooke, J. E., Eirich, R., Korczak, D. J., McArthur, B., & Madigan, S. (2020). Child and adolescent mental illness during COVID-19: A rapid review [Letter]. *Psychiatry Research*, *292*. <https://doi.org/10.1016/j.psychres.2020.113307>

Ramadhan, M. H. A., Putri, A. K., Melinda, D., Habibah, U., Fajriyah, U. N., Aini, S., Prananjaya, B. A., & Ikhsan, D. S. (2020). Children’s mental health in the time of COVID-19: How things stand and the aftermath [Article]. *Malaysian Journal of Medical Sciences*, *27*(5), 196-201. <https://doi.org/10.21315/mjms2020.27.5.15>

Ramos-Álvarez, O., Arufe-Giráldez, V., Cantarero-Prieto, D., & Ibáñez-García, A. (2021). Impact of SARS-CoV-2 lockdown on anthropometric parameters in children 11/12 years old [Article]. *Nutrients*, *13*(11). <https://doi.org/10.3390/nu13114174>

Reséndiz-Aparicio, J. C. (2021). How the COVID-19 contingency affects children [Article]. *Boletin Medico del Hospital Infantil de Mexico*, *78*(3), 216-224. <https://doi.org/10.24875/BMHIM.20000140>

Rezaeipour, M. (2021). COVID-19-related weight gain in school-aged children [Letter]. *International Journal of Endocrinology and Metabolism*, *19*(1). <https://doi.org/10.5812/IJEM.110634>

Rice, T., & Sher, L. (2021). The men's mental health perspective on adolescent suicide in the COVID-19 era. *Acta Neuropsychiatrica*, *33*(4), 178-181. <https://doi.org/10.1017/neu.2021.10>

Sá, C., Pombo, A., Luz, C., Rodrigues, L. P., & Cordovil, R. (2020). COVID-19 SOCIAL ISOLATION IN BRAZIL: EFFECTS ON THE PHYSICAL ACTIVITY ROUTINE OF FAMILIES WITH CHILDREN. *Rev Paul Pediatr*, *39*, e2020159. <https://doi.org/10.1590/1984-0462/2021/39/2020159>

Sadeghipour, H. R., Zar, A., Pakizeh, A., & Ramsbottom, R. (2021). Evaluation of health-related quality of life in physically active and physically inactive students during the COVID-19 pandemic in Iran. *Cities (London, England)*, *118*, 103367. <https://doi.org/10.1016/j.cities.2021.103367>

Saito, M., Kikuchi, Y., Lefor, A. K., & Hoshina, M. (2021). Mental health in Japanese children during school closures due to the COVID-19 [Article in Press]. *Pediatrics International*. <https://doi.org/10.1111/ped.14718>

Salussolia, A., Montalti, M., Marini, S., Rallo, F., Masini, A., Paterno, M., Agosta, M., Guaraldi, F., Dallolio, L., & Gori, D. (2021). Preliminary data on physical well-being of children and adolescents during the SARS-CoV-2 pandemic...14th European Public Health Conference (Virtual), Public health futures in a changing world, November 10-12, 2021. *European Journal of Public Health*, *31*, iii81-iii81. <https://search.ebscohost.com/login.aspx?direct=true&AuthType=sso&db=ccm&AN=153588560&site=ehost-live&custid=s3890005>

Salzano, G., Passanisi, S., Pira, F., Sorrenti, L., La Monica, G., Pajno, G. B., Pecoraro, M., & Lombardo, F. (2021). Quarantine due to the COVID-19 pandemic from the perspective of adolescents: the crucial role of technology [Article]. *Italian Journal of Pediatrics*, *47*(1). <https://doi.org/10.1186/s13052-021-00997-7>

Samji, H., Wu, J., Ladak, A., Vossen, C., Stewart, E., Dove, N., Long, D., & Snell, G. (2021). Review: Mental health impacts of the COVID-19 pandemic on children and youth – a systematic review [Article in Press]. *Child and Adolescent Mental Health*. <https://doi.org/10.1111/camh.12501>

Sarker, T., Sarkar, A., Rabbany, M. G., Barmon, M., Roy, R., Rahman, M. A., Hossain, K. Z., Hoque, F., & Asaduzzaman, M. (2021). Evaluation of preventive, supportive and awareness building measures among international students in China in response to COVID-19: a structural equation modeling approach. *Global health research and policy*, *6*(1), 10. <https://doi.org/10.1186/s41256-021-00192-5>

Schlichtiger, J., Brunner, S., Steffen, J., & Huber, B. C. (2020). Mental health impairment triggered by the COVID-19 pandemic in a sample population of German students [Article]. *Journal of Investigative Medicine*, *68*(8), 1394-1396. <https://doi.org/10.1136/jim-2020-001553>

Schmits, E., Dekeyser, S., Klein, O., Luminet, O., Yzerbyt, V., & Glowacz, F. (2021). Psychological distress among students in higher education: One year after the beginning of the COVID-19 pandemic [Article]. *International Journal of Environmental Research and Public Health*, *18*(14). <https://doi.org/10.3390/ijerph18147445>

Schnaiderman, D., Bailac, M., Borak, L., Comar, H., Eisner, A., Ferrari, A., Giannini, G., Risso, F., Vetere, C., & Garibotti, G. (2021). Psychological impact of COVID-19 lockdown in children and adolescents from San Carlos de Bariloche, Argentina: Parents' perspective. *Archivos Argentinos de Pediatria*, *119*(3), 170-176. <https://doi.org/10.5546/aap.2021.eng.170>

Sciberras, E., Patel, P., Stokes, M. A., Coghill, D., Middeldorp, C. M., Bellgrove, M. A., Becker, S. P., Efron, D., Stringaris, A., Faraone, S. V., Bellows, S. T., Quach, J., Banaschewski, T., McGillivray, J., Hutchinson, D., Silk, T. J., Melvin, G., Wood, A. G., Jackson, A., . . . Westrupp, E. (2022). Physical Health, Media Use, and Mental Health in Children and Adolescents With ADHD During the COVID-19 Pandemic in Australia [Article]. *Journal of attention disorders*, *26*(4), 549-562. <https://doi.org/10.1177/1087054720978549>

Sedumedi, C. M., Janssen, X., Reilly, J. J., Kruger, H. S., & Monyeki, M. A. (2021). Association between objectively determined physical activity levels and body composition in 6–8-year-old children from a black south african population: Bc–it study [Article]. *International Journal of Environmental Research and Public Health*, *18*(12). <https://doi.org/10.3390/ijerph18126453>

Shah, N., Karguppikar, M., Bhor, S., Ladkat, D., Khadilkar, V., & Khadilkar, A. (2021). Impact of lockdown for COVID-19 pandemic in Indian children and youth with type 1 diabetes from different socio-economic classes. *J Pediatr Endocrinol Metab*, *34*(2), 217-223. <https://doi.org/10.1515/jpem-2020-0460>

Sharma, V., Dhaliwal, S., & Singh, R. B. (2021). Psychological and Physical Implications of COVID-19 on School Children in India. In (Vol. 33, pp. 672-673): Sage Publications, Ltd.

Sharpe, D., Rajabi, M., Chileshe, C., Joseph, S. M., Sesay, I., Williams, J., & Sait, S. (2021). Mental health and wellbeing implications of the COVID-19 quarantine for disabled and disadvantaged children and young people: evidence from a cross-cultural study in Zambia and Sierra Leone [Article]. *BMC psychology*, *9*(1), 79. <https://doi.org/10.1186/s40359-021-00583-w>

Shaun, M. M. A., Nizum, M. W. R., Munny, S., Fayeza, F., Mali, S. K., Abid, M. T., & Hasan, A. R. (2021). Eating habits and lifestyle changes among higher studies students post-lockdown in Bangladesh: A web-based cross-sectional study. *Heliyon*, *7*(8), e07843. <https://doi.org/10.1016/j.heliyon.2021.e07843>

Shek, D. T. L. (2020). Chinese Adolescent Research Under COVID-19 [Editorial]. *Journal of Adolescent Health*, *67*(6), 733-734. <https://doi.org/10.1016/j.jadohealth.2020.09.011>

Shepherd, H. A., Evans, T., Gupta, S., McDonough, M. H., Doyle-Baker, P., Belton, K. L., Karmali, S., Pawer, S., Hadly, G., Pike, I., Adams, S. A., Babul, S., Emery, C. A., Yeates, K. O., Kopala Sibley, D. C., Schneider, K. J., Cowle, S., Fuselli, P., & Black, A. M. (2021). High school student-athletes' experiences with the COVID-19 pandemic: The impact on their physical activity engagement and mental health [Conference Abstract]. *Clinical Journal of Sport Medicine*, *31*(3), e126. <https://doi.org/10.1097/JSM.0000000000000932>

Shepherd, H. A., Evans, T., Gupta, S., McDonough, M. H., Doyle-Baker, P., Belton, K. L., Karmali, S., Pawer, S., Hadly, G., Pike, I., Adams, S. A., Babul, S., Yeates, K. O., Kopala-Sibley, D. C., Schneider, K. J., Cowle, S., Fuselli, P., Emery, C. A., & Black, A. M. (2021). The impact of COVID-19 on high school student-athlete experiences with physical activity, mental health, and social connection [Article]. *International Journal of Environmental Research and Public Health*, *18*(7). <https://doi.org/10.3390/ijerph18073515>

Shneor, E., Doron, R., Levine, J., Zimmerman, D. R., Benoit, J. S., Ostrin, L. A., & Gordon-Shaag, A. (2021). Objective behavioral measures in children before, during, and after the covid-19 lockdown in israel [Article]. *International Journal of Environmental Research and Public Health*, *18*(16). <https://doi.org/10.3390/ijerph18168732>

Siachpazidou, D. I., Kotsiou, O. S., Chatziparasidis, G., Papagiannis, D., Vavougios, G. D., Gogou, E., Stavrou, V. T., & Gourgoulianis, K. I. (2021). Action and reaction of pre-primary and primary school-age children to restrictions during covid-19 pandemic in greece [Article]. *Journal of Personalized Medicine*, *11*(6). <https://doi.org/10.3390/jpm11060451>

Simpson, L., Williams, M., Batchford, C., Davison, A., Dawson, P., Holt, A., Ghumra, S., Singh, A., Raju, K., Alkotamy, M., Haque, M., Hailston, L., Packham, A., O'Sullivan, R., Kelly, L., Brace, L., Thompson, J., Rapson, A., Danaher, S., . . . Hartshorn, S. (2021). Bringing our heeadsss together-prioritising the voices of children and young people Collaborators [Conference Abstract]. *Archives of Disease in Childhood*, *106*(SUPPL 1), A208-A209. <https://doi.org/10.1136/archdischild-2021-rcpch.363>

Sinaei, R., Pezeshki, S., Yazdani, M., Sabzevari, F., & Hassas Yeganeh, M. (2021). The psychological consequences of covid-19 on children’s world [Letter]. *Iranian Journal of Child Neurology*, *15*(2), 87-89. <https://doi.org/10.22037/ijcn.v15i1.31668>

Singh, A., Shah, N., Mbeledogu, C., & Garstang, J. (2021). Child wellbeing in the United Kingdom following the COVID-19 lockdowns [Review]. *Paediatrics and Child Health (United Kingdom)*, *31*(12), 445-448. <https://doi.org/10.1016/j.paed.2021.09.004>

Singh, S., & Balhara, Y. (2021). 'Screen-time' for children and adolescents in COVID-19 times: Need to have the contextually informed perspective [Review]. *Indian Journal of Psychiatry*, *63*(2), 192-195. <https://doi.org/10.4103/psychiatry.IndianJPsychiatry_646_20>

Slomski, A. (2021). Pediatric Depression and Anxiety Doubled during the Pandemic [Note]. *JAMA - Journal of the American Medical Association*, *326*(13), 1246. <https://doi.org/10.1001/jama.2021.16374>

Spitzer, M. (2021). Open schools! Weighing the effects of viruses and lockdowns on children [Article]. *Trends in Neuroscience and Education*, *22*. <https://doi.org/10.1016/j.tine.2021.100151>

Storz, M. A. (2020). The COVID-19 pandemic: An unprecedented tragedy in the battle against childhood obesity [Article]. *Korean Journal of Pediatrics*, *63*(12), 477-482. <https://doi.org/10.3345/cep.2020.01081>

Štveráková, T., Jačisko, J., Busch, A., Šafářová, M., Kolář, P., & Kobesová, A. (2021). The impact of COVID-19 on physical activity of Czech children. *PLoS ONE*, *16*(7), 14. <https://doi.org/http://dx.doi.org/10.1371/journal.pone.0254244>

Suhua, X., Zi, Y., & Li, Z. (2021). Physical Activity, Screen Time, and Mood Disturbance Among Chinese Adolescents During COVID-19. *Journal of Psychosocial Nursing & Mental Health Services*, *59*(4), 14-20. <https://doi.org/10.3928/02793695-20201104-04>

Surekha, B. C., Karanati, K., Venkatesan, K., Sreelekha, B. C., & Kumar, V. D. (2021). E-Learning During COVID-19 Pandemic: A Surge in Childhood Obesity. *Indian journal of otolaryngology and head and neck surgery : official publication of the Association of Otolaryngologists of India*, 1-7. <https://doi.org/10.1007/s12070-021-02750-2>

Susilowati, I. H., Nugraha, S., Alimoeso, S., & Hasiholan, B. P. (2021). Screen Time for Preschool Children: Learning from Home during the COVID-19 Pandemic [Article]. *Global Pediatric Health*, *8*. <https://doi.org/10.1177/2333794X211017836>

Sutter, E. N., Francis, L. S., Francis, S. M., Lench, D. H., Nemanich, S. T., Krach, L. E., Sukal-Moulton, T., & Gillick, B. T. (2021). Disrupted Access to Therapies and Impact on Well-Being During the COVID-19 Pandemic for Children With Motor Impairment and Their Caregivers [Article]. *American Journal of Physical Medicine & Rehabilitation*, *100*(9), 821-830. <https://doi.org/10.1097/PHM.0000000000001818>

Szpunar, G., Cannoni, E., & Di Norcia, A. (2021). La didattica a distanza durante il lockdown in Italia: il punto di vista delle famiglie [Distance learning during the lockdown in Italy: The point of view of families]. *Journal of Educational, Cultural and Psychological Studies*, *23*, 137-155. <https://www.proquest.com/scholarly-journals/la-didattica-distanza-durante-il-lockdown-italia/docview/2594717034/se-2?accountid=164166>

http://sfx.csids.edu.hk/cihe??url_ver=Z39.88-2004&rft_val_fmt=info:ofi/fmt:kev:mtx:journal&genre=article&sid=ProQ:ProQ%3Apsycinfo&atitle=La+didattica+a+distanza+durante+il+lockdown+in+Italia%3A+il+punto+di+vista+delle+famiglie&title=Journal+of+Educational%2C+Cultural+and+Psychological+Studies&issn=20377932&date=2021-01-01&volume=23&issue=&spage=137&au=Szpunar%2C+Giordana%3BCannoni%2C+Eleonora%3BDi+Norcia%2C+Anna&isbn=&jtitle=Journal+of+Educational%2C+Cultural+and+Psychological+Studies&btitle=&rft_id=info:eric/2021-68503-005&rft_id=info:doi/

Szwarcwald, C. L., Malta, D. C., Barros, M. B. A., Júnior, P. R. B. S., Romero, D., de Almeida, W. D. S., Damacena, G. N., Werneck, A. O., da Silva, D. R. P., Lima, M. G., Gomes, C. S., Azevedo, L. O., Ferreira, A. P. S., Gracie, R., & de Pina, M. F. (2021). Associations of sociodemographic factors and health behaviors with the emotional well-being of adolescents during the covid-19 pandemic in Brazil [Article]. *International Journal of Environmental Research and Public Health*, *18*(11). <https://doi.org/10.3390/ijerph18116160>

Tandon, P. S., Zhou, C., Johnson, A. M., Gonzalez, E. S., & Kroshus, E. (2021). Association of children's physical activity and screen time with mental health during the covid-19 pandemic [Article]. *JAMA Network Open*, *4*(10). <https://doi.org/10.1001/jamanetworkopen.2021.27892>

Tardif-Grenier, K., Archambault, I., Dupéré, V., Marks, A. K., & Olivier, E. (2021). Canadian Adolescents’ Internalized Symptoms in Pandemic Times: Association with Sociodemographic Characteristics, Confinement Habits, and Support [Article]. *Psychiatric Quarterly*, *92*(3), 1309-1325. <https://doi.org/10.1007/s11126-021-09895-x>

Thakur, K., Kumar, N., & Sharma, N. R. (2020). Effect of the Pandemic and Lockdown on Mental Health of Children [Letter]. *Indian Journal of Pediatrics*, *87*(7), 552. <https://doi.org/10.1007/s12098-020-03308-w>

Thomas, J., Bowes, N., Meyers, R., & Thirlaway, K. (2021). Mental well-being and physical activity of young people experiencing homelessness before and during COVID-19 lockdown: A longitudinal study [Article]. *Mental Health and Physical Activity*, *21*. <https://doi.org/10.1016/j.mhpa.2021.100407>

Toppe, T., Stengelin, R., Schmidt, L. S., Amini, N., & Schuhmacher, N. (2021). Explaining Variation in Parents' and Their Children's Stress During COVID-19 Lockdowns. *Frontiers in Psychology*, *12*, 645266. <https://doi.org/10.3389/fpsyg.2021.645266>

Tornaghi, M., Lovecchio, N., Vandoni, M., Chirico, A., & Codella, R. (2021). Physical activity levels across COVID-19 outbreak in youngsters of Northwestern Lombardy [Article]. *The Journal of sports medicine and physical fitness*, *61*(7), 971-976. <https://doi.org/10.23736/S0022-4707.20.11600-1>

Tse, W. W. Y., & Kwan, M. Y. W. (2021). Impacts of the covid-19 pandemic on the physical and mental health of children [Editorial]. *Hong Kong Medical Journal*, *27*(3), 175-176. <https://doi.org/10.12809/hkmj215118>

Tso, W. W. Y., Wong, R. S., Tung, K. T. S., Rao, N., Fu, K. W., Yam, J. C. S., Chua, G. T., Chen, E. Y. H., Lee, T. M. C., Chan, S. K. W., Wong, W. H. S., Xiong, X., Chui, C. S., Li, X., Wong, K., Leung, C., Tsang, S. K. M., Chan, G. C. F., Tam, P. K. H., & Chan, K. L. (2022). Vulnerability and resilience in children during the COVID-19 pandemic. *European Child & Adolescent Psychiatry*, *31*(1), 161-176. <https://doi.org/10.1007/s00787-020-01680-8>

Valadez, M. L. D., Rodríguez-Naveiras, E., Castellanos-Simons, D., López-Aymes, G., Aguirre, T., Flores, J. F., & Borges, Á. (2020). Physical Activity and Well-Being of High Ability Students and Community Samples During the COVID-19 Health Alert. *Front Psychol*, *11*, 606167. <https://doi.org/10.3389/fpsyg.2020.606167>

Valenzise, M., D'Amico, F., Cucinotta, U., Lugarà, C., Zirilli, G., Zema, A., Wasniewska, M., & Pajno, G. B. (2021). The lockdown effects on a pediatric obese population in the COVID-19 era. *Italian Journal of Pediatrics*, *47*(1), 209. <https://doi.org/10.1186/s13052-021-01142-0>

Vall-Roqué, H., Andrés, A., & Saldaña, C. (2021). The impact of COVID-19 lockdown on social network sites use, body image disturbances and self-esteem among adolescent and young women [Article]. *Progress in Neuro-Psychopharmacology and Biological Psychiatry*, *110*. <https://doi.org/10.1016/j.pnpbp.2021.110293>

Ventura, P. S., Ortigoza, A. F., Castillo, Y., Bosch, Z., Casals, S., Girbau, C., Siurana, J. M., Arce, A., Torres, M., Herrero, F. J., Sebastiani, G., & Andreu-Fernández, V. (2021). Children's Health Habits and COVID-19 Lockdown in Catalonia: Implications for Obesity and Non-Communicable Diseases. *Nutrients*, *13*(5), 1657. <https://doi.org/10.3390/nu13051657>

Vuković, J., Matić, R. M., Milovanović, I. M., Maksimović, N., Krivokapić, D., & Pišot, S. (2021). Children's Daily Routine Response to COVID-19 Emergency Measures in Serbia [Article]. *Frontiers in Pediatrics*, *9*. <https://doi.org/10.3389/fped.2021.656813>

Vyjayanthi, N. V., Banerjee, D., & Sathyanarayana Rao, T. S. (2020). The silent victims of the pandemic: Children and adolescents during the COVID-19 crisis [Editorial]. *Journal of Indian Association for Child and Adolescent Mental Health*, *16*(3), 17-31. <https://www.embase.com/search/results?subaction=viewrecord&id=L2004898894&from=export>

Wahl-Alexander, Z., & Camic, C. L. (2021). Impact of COVID-19 on School-Aged Male and Female Health-Related Fitness Markers [Article]. *Pediatric Exercise Science*, *33*(2), 61-64. <https://doi.org/10.1123/pes.2020-0208>

Wang, G., Zhang, Y., Zhao, J., Zhang, J., & Jiang, F. (2020). Mitigate the effects of home confinement on children during the COVID-19 outbreak. *The Lancet*, *395*(10228), 945-947. <https://doi.org/http://dx.doi.org/10.1016/S0140-6736(20)30547-X>

Wang, L., Hao, Y., Chen, L., Zhang, Y. W., Deng, H. Z., Ke, X. Y., Wang, J. H., Li, F., Hou, Y., Xie, X. H., Xu, Q., Wang, X., Guan, H. Y., Wang, W. J., Shen, J. N., Li, F., Qian, Y., Zhang, L. L., Shi, X. M., . . . Li, T. Y. (2021). Psychological and behavioral functioning of children and adolescents during long-term home-schooling [Article]. *Zhonghua yu fang yi xue za zhi [Chinese journal of preventive medicine]*, *55*(9), 1059-1066. <https://doi.org/10.3760/cma.j.cn112150-20210602-00533>

Wang, L., Zhang, Y., Chen, L., Wang, J., Jia, F., Li, F., Froehlich, T. E., Hou, Y., Hao, Y., Shi, Y., Deng, H., Zhang, J., Huang, L., Xie, X., Fang, S., Xu, L., Xu, Q., Guan, H., Wang, W., . . . Li, T. (2021). Psychosocial and behavioral problems of children and adolescents in the early stage of reopening schools after the COVID-19 pandemic: a national cross-sectional study in China [Article]. *Translational Psychiatry*, *11*(1). <https://doi.org/10.1038/s41398-021-01462-z>

Wang, M. T., Scanlon, C. L., Hua, M., Belmont, A. M., Zhang, A. L., & Toro, J. D. (2021). Social Distancing and Adolescent Psychological Well-Being: The Role of Practical Knowledge and Exercise [Article in Press]. *Academic Pediatrics*. <https://doi.org/10.1016/j.acap.2021.10.008>

Wang, P., Sun, X., Li, W., Wang, Z., He, S., Zhai, F., Xin, Y., Pan, L., Wang, G., Jiang, F., & Chen, J. (2021). Mental Health of Parents and Preschool-Aged Children During the COVID-19 Pandemic: The Mediating Role of Harsh Parenting and Child Sleep Disturbances [Article]. *Frontiers in Psychiatry*, *12*. <https://doi.org/10.3389/fpsyt.2021.746330>

Weatherspoon, B. L. (2021). *Promoting physical activity breaks in Clairton city school district sixth grade classrooms* (Publication Number AAI28370802) [Ed.D., University of Pittsburgh]. APA PsycInfo®. <https://www.proquest.com/dissertations-theses/promoting-physical-activity-breaks-clairton-city/docview/2509688503/se-2?accountid=164166>

http://sfx.csids.edu.hk/cihe??url_ver=Z39.88-2004&rft_val_fmt=info:ofi/fmt:kev:mtx:dissertation&genre=dissertations&sid=ProQ:APA+PsycInfo%C2%AE&atitle=&title=Promoting+physical+activity+breaks+in+Clairton+city+school+district+sixth+grade+classrooms&issn=&date=2021-01-01&volume=&issue=&spage=&au=Weatherspoon%2C+Britnee+L.&isbn=979-8557076234&jtitle=&btitle=&rft_id=info:eric/2021-27918-022&rft_id=info:doi/

Welling, M. S., Abawi, O., Van Den Eynde, E., Van Rossum, E. F. C., Halberstadt, J., Brandsma, A. E., Kleinendorst, L., Van Den Akker, E. L. T., & Van Der Voorn, B. (2021). Impact of the COVID-19 pandemic and related lockdown measures on lifestyle behaviors and wellbeing in children and adolescents with severe obesity [Article in Press]. *Obesity Facts*. <https://doi.org/10.1159/000520718>

Wickramasinghe, K. (2021). COVID-19: Impacts on child and adolescent obesity and health [Conference Abstract]. *Obesity Facts*, *14*(SUPPL 1), 1. <https://doi.org/10.1159/000515911>

Wright, L. J., Williams, S. E., & Veldhuijzen van Zanten, J. (2021). Physical Activity Protects Against the Negative Impact of Coronavirus Fear on Adolescent Mental Health and Well-Being During the COVID-19 Pandemic. *Front Psychol*, *12*, 580511. <https://doi.org/10.3389/fpsyg.2021.580511>

Xiang, M., Yamamoto, S., & Mizoue, T. (2020). Depressive symptoms in students during school closure due to COVID-19 in Shanghai [Letter]. *Psychiatry and Clinical Neurosciences*, *74*(12), 664-666. <https://doi.org/10.1111/pcn.13161>

Xiang, M., Zhang, Z., & Kuwahara, K. (2020). Impact of COVID-19 pandemic on children and adolescents' lifestyle behavior larger than expected. *Progress in Cardiovascular Diseases*, *63*(4), 531-532. <https://doi.org/10.1016/j.pcad.2020.04.013>

Ye, J. (2020). Pediatric Mental and Behavioral Health in the Period of Quarantine and Social Distancing With COVID-19. *JMIR pediatrics and parenting*, *3*(2), e19867. <https://doi.org/10.2196/19867>

Yuan, Y. Q., Ding, J. N., Bi, N., Wang, M. J., Zhou, S. C., Wang, X. L., Zhang, S. H., Liu, Y., & Roswal, G. (2021). Physical activity and sedentary behaviour among children and adolescents with intellectual disabilities during the covid‐19 lockdown in china. *Journal of Intellectual Disability Research*. <https://doi.org/http://dx.doi.org/10.1111/jir.12898>

Zhai, X., Zeng, J., Eshak, E. S., Zhang, Y., Yang, M., Di, L., Xiang, B., & Cao, J. (2021). The influencing factors of sleep quality among Chinese junior and senior high school adolescents during the COVID-19 pandemic [Review]. *Journal of Tropical Pediatrics*, *67*(4). <https://doi.org/10.1093/tropej/fmab069>

Zhang, J., Seo, D. C., Kolbe, L., Lee, A., Middlestadt, S., Zhao, W., & Huang, S. (2011). Comparison of overweight, weight perception, and weight-related practices among high school students in three large Chinese cities and two large U.S. cities. *J Adolesc Health*, *48*(4), 366-372. <https://doi.org/10.1016/j.jadohealth.2010.07.015>

Zhang, J., Zhou, Z., & Zhang, W. (2021). Intervention effect of research-based psychological counseling on adolescents mental health during the covid-19 epidemic [Article]. *Psychiatria Danubina*, *33*(2), 209-216. <https://doi.org/10.24869/psyd.2021.209>

Zhang, X. (2021). Association of COVID-19 Mitigation Measures with Cardiorespiratory Fitness and Body Mass Index among Children in Austria [Note]. *JAMA Network Open*, *4*(8). <https://doi.org/10.1001/jamanetworkopen.2021.21965>

Zhou, J., Yuan, X., Qi, H., Liu, R., Li, Y., Huang, H., Chen, X., & Wang, G. (2020). Prevalence of depression and its correlative factors among female adolescents in China during the coronavirus disease 2019 outbreak [Article]. *Globalization and Health*, *16*(1). <https://doi.org/10.1186/s12992-020-00601-3>

Zhu, S., Zhuang, Y., & Ip, P. (2021). Impacts on children and adolescents’ lifestyle, social support and their association with negative impacts of the covid‐19 pandemic [Article]. *International Journal of Environmental Research and Public Health*, *18*(9). <https://doi.org/10.3390/ijerph18094780>

S**upplementary Table 4. NOS scores for included articles.**

| Authors | NOS | | | | | | | | | |
| --- | --- | --- | --- | --- | --- | --- | --- | --- | --- | --- |
|  | 1 | 2 | 3 | 4 | 5 | 6 | 7 | 8 | 9 | Total |
| Abid *et al.* (2021) | ★ | ★ | ★ |  | ★ | ★ | ★ | ★ |  | 7 |
| Acosta *et al.* (2021) | ★ |  | ★ |  |  |  |  |  | ★ | 3 |
| Aguilar-Farias *et al.* (2020) | ★ | ★ | ★ |  | ★ | ★ | ★ | ★ | ★ | 8 |
| Alonso-Martínez *et al.* (2021) | ★ | ★ | ★ |  | ★ | ★ | ★ | ★ | ★ | 8 |
| Berasategi et al. (2021) | ★ |  | ★ |  |  |  |  | ★ | ★ | 4 |
| Breidokienè *et al.* (2021) | ★ | ★ | ★ |  | ★ | ★ | ★ | ★ | ★ | 8 |
| Bringolf-Isler *et al.* (2021) | ★ | ★ | ★ |  | ★ | ★ | ★ | ★ |  | 7 |
| Brzęk *et al.* (2021) | ★ | ★ | ★ |  | ★ | ★ | ★ | ★ |  | 7 |
| Burdzovic Andreas & Brunborg (2021) | ★ | ★ | ★ |  | ★ | ★ | ★ | ★ | ★ | 8 |
| Chen *et al.* (2021) | ★ | ★ | ★ |  | ★ | ★ | ★ | ★ |  | 7 |
| Chen *et al.* (2022) | ★ | ★ | ★ |  | ★ | ★ | ★ | ★ |  | 7 |
| Chi *et al.* (2021) | ★ |  | ★ |  | ★ | ★ | ★ | ★ |  | 6 |
| Dragun *et al.* (2020) | ★ | ★ | ★ |  | ★ | ★ | ★ | ★ | ★ | 8 |
| Francisco *et al.* (2020) | ★ | ★ | ★ |  | ★ | ★ | ★ | ★ |  | 7 |
| Ghanamah & Eghbaria-Ghanamah (2020) | ★ | ★ | ★ |  | ★ | ★ | ★ | ★ | ★ | 8 |
| Ghorbani *et al.* (2021) | ★ | ★ | ★ |  | ★ | ★ | ★ | ★ | ★ | 8 |
| Gilbert *et al.* (2021) | ★ | ★ | ★ |  | ★ | ★ | ★ | ★ |  | 7 |
| Hossain *et al.* (2021) | ★ | ★ | ★ |  | ★ | ★ | ★ | ★ | ★ | 8 |
| Hyunshik *et al.* (2021) | ★ | ★ | ★ |  | ★ | ★ | ★ | ★ | ★ | 8 |
| Ishimoto *et al.* (2022) | ★ | ★ | ★ |  | ★ | ★ | ★ | ★ | ★ | 8 |
| Jackson *et al.* (2021) | ★ | ★ | ★ |  | ★ | ★ | ★ | ★ | ★ | 8 |
| Jauregui *et al.* (2021) | ★ | ★ | ★ |  | ★ | ★ | ★ | ★ | ★ | 8 |
| Jester & Kang (2021) | ★ | ★ | ★ |  | ★ | ★ | ★ | ★ | ★ | 8 |
| Jovanović *et al.* (2021) | ★ | ★ | ★ |  | ★ | ★ | ★ | ★ |  | 7 |
| Kang *et al.* (2021) | ★ | ★ | ★ |  | ★ | ★ | ★ | ★ | ★ | 8 |
| Kuhn *et al.* (2022) | ★ | ★ | ★ |  | ★ | ★ | ★ | ★ |  | 7 |
| Laurier *et al.* (2021) | ★ | ★ | ★ |  | ★ | ★ | ★ | ★ | ★ | 8 |
| D. J. Lee *et al.* (2021) | ★ |  | ★ |  |  |  |  |  |  | 2 |
| S. -M. Lee *et al.* (2021) | ★ |  | ★ |  | ★ | ★ | ★ | ★ |  | 6 |
| Lim *et al.* (2021) | ★ | ★ | ★ |  | ★ | ★ | ★ | ★ | ★ | 8 |
| Liu *et al.* (2021) | ★ |  | ★ |  |  |  |  | ★ | ★ | 4 |
| Lu *et al.* (2021) | ★ | ★ | ★ |  | ★ | ★ | ★ | ★ | ★ | 8 |
| Łuszczki *et al.* (2021) | ★ | ★ | ★ |  | ★ | ★ | ★ | ★ | ★ | 8 |
| Mâsse *et al.* (2021) | ★ |  | ★ |  | ★ | ★ | ★ | ★ |  | 6 |
| McArthur *et al.* (2021) | ★ | ★ | ★ |  | ★ | ★ | ★ | ★ | ★ | 8 |
| Medrano *et al.* (2021) | ★ | ★ | ★ |  | ★ | ★ | ★ | ★ | ★ | 8 |
| Min *et al.* (2021) | ★ |  | ★ |  |  |  | ★ | ★ |  | 4 |
| Mitra *et al.* (2021) | ★ |  | ★ |  | ★ | ★ | ★ | ★ |  | 6 |
| Mzadi *et. al.* (2022) | ★ | ★ | ★ |  | ★ | ★ | ★ | ★ | ★ | 8 |
| Ren *et al.* (2021) | ★ | ★ | ★ |  | ★ | ★ | ★ | ★ | ★ | 8 |
| Tulchin *et al.* (2021) | ★ |  | ★ |  | ★ | ★ | ★ | ★ |  | 6 |
| Wang *et al.* (2021) | ★ | ★ | ★ |  | ★ | ★ | ★ | ★ | ★ | 8 |
| Wunsch *et al.* (2021) | ★ | ★ | ★ |  | ★ | ★ | ★ | ★ |  | 7 |
| Zhang *et al.* (2020) | ★ | ★ | ★ |  | ★ | ★ | ★ | ★ | ★ | 8 |

*See checklist of [NOS of cohort studies](https://www.ncbi.nlm.nih.gov/books/NBK115843/bin/appe-fm3.pdf)

**References of Supplementary Table 4.**

Abid, R., Ammar, A., Maaloul, R., Souissi, N., & Hammouda, O. (2021). Effect of COVID-19-related home confinement on sleep quality, screen time and physical activity in tunisian boys and girls: A survey [Article]. *International Journal of Environmental Research and Public Health*, *18*(6), 1-12. <https://doi.org/10.3390/ijerph18063065>

Acosta, D., Fujii, Y., Joyce-Beaulieu, D., Jacobs, K. D., Maurelli, A. T., Nelson, E. J., & McKune, S. L. (2021). Psychosocial health of k-12 students engaged in emergency remote education and in-person schooling: A cross-sectional study [Article]. *International Journal of Environmental Research and Public Health*, *18*(16). <https://doi.org/10.3390/ijerph18168564>

Aguilar-Farias, N., Toledo-Vargas, M., Miranda-Marquez, S., Cortinez-O'Ryan, A., Cristi-Montero, C., Rodriguez-Rodriguez, F., Martino-Fuentealba, P., Okely, A. D., & Del Pozo Cruz, B. (2020). Sociodemographic Predictors of Changes in Physical Activity, Screen Time, and Sleep among Toddlers and Preschoolers in Chile during the COVID-19 Pandemic. *International Journal of Environmental Research and Public Health*, *18*(1). <https://doi.org/10.3390/ijerph18010176>

Alonso-Martínez, A. M., Ramírez-Vélez, R., García-Alonso, Y., Izquierdo, M., & García-Hermoso, A. (2021). Physical activity, sedentary behavior, sleep and self-regulation in spanish preschoolers during the COVID-19 lockdown [Article]. *International Journal of Environmental Research and Public Health*, *18*(2), 1-8. <https://doi.org/10.3390/ijerph18020693>

Berasategi Sancho, N., Idoiaga Mondragon, N., Dosil Santamaria, M., & Eiguren Munitis, A. (2021). The Well-being of children in lock-down: Physical, emotional, social and academic impact. *Children & Youth Services Review*, *127*, N.PAG-N.PAG. <https://doi.org/10.1016/j.childyouth.2021.106085>

Breidokienė, R., Jusienė, R., Urbonas, V., Praninskienė, R., & Girdzijauskienė, S. (2021). Sedentary Behavior among 6-14-Year-Old Children during the COVID-19 Lockdown and Its Relation to Physical and Mental Health. *Healthcare (Basel, Switzerland)*, *9*(6). <https://doi.org/10.3390/healthcare9060756>

Bringolf-Isler, B., Hänggi, J., Kayser, B., L Suggs, S., Dössegger, A., & Probst-Hensch, N. (2021). COVID-19 pandemic and health related quality of life in primary school children in Switzerland: a repeated cross-sectional study [Article]. *Swiss Medical Weekly*, *151*, w30071. <https://doi.org/10.4414/smw.2021.w30071>

Brzęk, A., Strauss, M., Sanchis-Gomar, F., & Leischik, R. (2021). Physical activity, screen time, sedentary and sleeping habits of polish preschoolers during the covid-19 pandemic and who’s recommendations: An observational cohort study [Article]. *International Journal of Environmental Research and Public Health*, *18*(21). <https://doi.org/10.3390/ijerph182111173>

Burdzovic Andreas, J., & Brunborg, G. S. (2021). Self-reported Mental and Physical Health among Norwegian Adolescents before and during the COVID-19 Pandemic [Article in Press]. *JAMA Network Open*. <https://doi.org/10.1001/jamanetworkopen.2021.21934>

Chen, X., Qi, H., Liu, R., Feng, Y., Li, W., Xiang, M., Cheung, T., Jackson, T., Wang, G., & Xiang, Y. T. (2021). Depression, anxiety and associated factors among Chinese adolescents during the COVID-19 outbreak: a comparison of two cross-sectional studies [Article]. *Translational Psychiatry*, *11*(1). <https://doi.org/10.1038/s41398-021-01271-4>

Chen, Y., Osika, W., Henriksson, G., Dahlstrand, J., & Friberg, P. (2022). Impact of COVID-19 pandemic on mental health and health behaviors in Swedish adolescents [Article]. *Scandinavian journal of public health*, *50*(1), 26-32. <https://doi.org/10.1177/14034948211021724>

Chi, X., Liang, K., Chen, S. T., Huang, Q., Huang, L., Yu, Q., Jiao, C., Guo, T., Stubbs, B., Hossain, M. M., Yeung, A., Kong, Z., & Zou, L. (2021). Mental health problems among Chinese adolescents during the COVID-19: The importance of nutrition and physical activity. *Int J Clin Health Psychol*, *21*(3), 100218. <https://doi.org/10.1016/j.ijchp.2020.100218>

Dragun, R., Veček, N. N., Marendić, M., Pribisalić, A., Đivić, G., Cena, H., Polašek, O., & Kolčić, I. (2020). Have Lifestyle Habits and Psychological Well-Being Changed among Adolescents and Medical Students Due to COVID-19 Lockdown in Croatia? *Nutrients*, *13*(1). <https://doi.org/10.3390/nu13010097>

Francisco, R., Pedro, M., Delvecchio, E., Espada, J. P., Morales, A., Mazzeschi, C., & Orgilés, M. (2020). Psychological Symptoms and Behavioral Changes in Children and Adolescents During the Early Phase of COVID-19 Quarantine in Three European Countries [Article]. *Frontiers in Psychiatry*, *11*. <https://doi.org/10.3389/fpsyt.2020.570164>

Ghanamah, R., & Eghbaria-Ghanamah, H. (2021). Impact of covid-19 pandemic on behavioral and emotional aspects and daily routines of Arab israeli children [Article]. *International Journal of Environmental Research and Public Health*, *18*(6), 1-19. <https://doi.org/10.3390/ijerph18062946>

Ghorbani, S., Afshari, M., Eckelt, M., Dana, A., & Bund, A. (2021). Associations between Physical Activity and Mental Health in Iranian Adolescents during the COVID-19 Pandemic: An Accelerometer-Based Study. *Children*, *8*(11), 1-11. <https://doi.org/10.3390/children8111022>

Gilbert, A. S., Schmidt, L., Beck, A., Kepper, M. M., Mazzucca, S., & Eyler, A. (2021). Associations of physical activity and sedentary behaviors with child mental well-being during the COVID-19 pandemic [Article]. *BMC Public Health*, *21*(1), 1770. <https://doi.org/10.1186/s12889-021-11805-6>

Hossain, M. S., Deeba, I. M., Hasan, M., Kariippanon, K. E., Chong, K. H., Cross, P. L., Ferdous, S., & Okely, A. D. (2021). International study of 24-h movement behaviors of early years (SUNRISE): a pilot study from Bangladesh [Article]. *Pilot and Feasibility Studies*, *7*(1). <https://doi.org/10.1186/s40814-021-00912-1>

Hyunshik, K., Jiameng, M., Sunkyoung, L., & Ying, G. (2021). Change in Japanese children's 24-hour movement guidelines and mental health during the COVID-19 pandemic [Article]. *Scientific reports*, *11*(1), 22972. <https://doi.org/10.1038/s41598-021-01803-4>

Ishimoto, Y., Yamane, T., Matsumoto, Y., Takizawa, Y., & Kobayashi, K. (2022). The impact of gender differences, school adjustment, social interactions, and social activities on emotional and behavioral reactions to the COVID-19 pandemic among Japanese school children. *SSM Ment Health*, *2*, 100077. <https://doi.org/10.1016/j.ssmmh.2022.100077>

Jackson, A., Lanigan, J., & Weaver, R. H. (2021). Health behavior changes during COVID-19: Physical activity, diet intake, and social isolation [Conference Abstract]. *Psychosomatic Medicine*, *83*(7), A35. <https://doi.org/10.1097/PSY.0000000000000997>

Jáuregui, A., Argumedo, G., Medina, C., Bonvecchio-Arenas, A., Romero-Martínez, M., & Okely, A. D. (2021). Factors associated with changes in movement behaviors in toddlers and preschoolers during the COVID-19 pandemic: A national cross-sectional study in Mexico [Article]. *Preventive Medicine Reports*, *24*. <https://doi.org/10.1016/j.pmedr.2021.101552>

Jester, N., & Kang, P. (2021). COVID-19 pandemic: Is teenagers' health in crisis? An investigation into the effects of COVID-19 on self-reported mental and physical health of teenagers in secondary education. *Public health in practice (Oxford, England)*, *2*, 100099. <https://doi.org/10.1016/j.puhip.2021.100099>

Jovanović, G. K., Zubalj, N. D., Majanović, S. K., Rahelić, D., Rahelić, V., Lončar, J. V., & Žeželj, S. P. (2021). The outcome of COVID-19 lockdown on changes in body mass index and lifestyle among croatian schoolchildren: A cross-sectional study [Article]. *Nutrients*, *13*(11). <https://doi.org/10.3390/nu13113788>

Kang, S., Sun, Y., Zhang, X., Sun, F., Wang, B., & Zhu, W. (2021). Is physical activity associated with mental health among chinese adolescents during isolation in COVID-19 pandemic? [Article]. *Journal of Epidemiology and Global Health*, *11*(1), 26-33. <https://doi.org/10.2991/JEGH.K.200908.001>

Kuhn, A. P., Kowalski, A. J., Wang, Y., Deitch, R., Selam, H., Rahmaty, Z., Black, M. M., & Hager, E. R. (2022). On the move or barely moving? Age-related changes in physical activity, sedentary, and sleep behaviors by weekday/weekend following pandemic control policies [Article]. *International Journal of Environmental Research and Public Health*, *19*(1). <https://doi.org/10.3390/ijerph19010286>

Laurier, C., Pascuzzo, K., & Beaulieu, G. (2021). Uncovering the personal and environmental factors associated with youth mental health during the COVID-19 pandemic: The pursuit of sports and physical activity as a protective factor. *Traumatology*, *27*(4), 354-364. <https://doi.org/http://dx.doi.org/10.1037/trm0000342>

Lee, D. J., So, W. Y., & Lee, S. M. (2021). The relationship between korean adolescents’ sports participation, internal health locus of control, and wellness during covid-19 [Article]. *International Journal of Environmental Research and Public Health*, *18*(6), 1-13. <https://doi.org/10.3390/ijerph18062950>

Lee, S.-M., So, W.-Y., & Youn, H.-S. (2021). Importance-Performance Analysis of Health Perception among Korean Adolescents during the COVID-19 Pandemic. *International Journal of Environmental Research and Public Health*, *18*(3). <https://doi.org/10.3390/ijerph18031280>

Lim, M. T. C., Ramamurthy, M. B., Aishworiya, R., Rajgor, D. D., Tran, A. P., Hiriyur, P., Kunaseelan, S., Jabri, M., & Goh, D. Y. T. (2021). School closure during the coronavirus disease 2019 (COVID-19) pandemic – Impact on children's sleep [Article]. *Sleep Medicine*, *78*, 108-114. <https://doi.org/10.1016/j.sleep.2020.12.025>

Liu, Q., Zhou, Y., Xie, X., Xue, Q., Zhu, K., Wan, Z., Wu, H., Zhang, J., & Song, R. (2021). The prevalence of behavioral problems among school-aged children in home quarantine during the COVID-19 pandemic in china [Article]. *Journal of Affective Disorders*, *279*. <https://doi.org/10.1016/j.jad.2020.10.008>

Lu, C., Chi, X., Liang, K., Chen, S.-T., Huang, L., Guo, T., Jiao, C., Yu, Q., Veronese, N., Soares, F. C., Grabovac, I., Yeung, A., & Zou, L. (2020). Moving More and Sitting Less as Healthy Lifestyle Behaviors are Protective Factors for Insomnia, Depression, and Anxiety Among Adolescents During the COVID-19 Pandemic. *Psychology research and behavior management*, *13*, 1223-1233. <https://doi.org/10.2147/PRBM.S284103>

Łuszczki, E., Bartosiewicz, A., Pezdan-śliż, I., Kuchciak, M., Jagielski, P., Oleksy, Ł., Stolarczyk, A., & Dereń, K. (2021). Children’s eating habits, physical activity, sleep, and media usage before and during COVID-19 pandemic in Poland [Article]. *Nutrients*, *13*(7). <https://doi.org/10.3390/nu13072447>

Mâsse, L. C., Edache, I. Y., Pitblado, M., & Hutchison, S. M. (2021). The impact of financial and psychological wellbeing on children’s physical activity and screen-based activities during the COVID-19 pandemic [Article]. *International Journal of Environmental Research and Public Health*, *18*(16). <https://doi.org/10.3390/ijerph18168694>

McArthur, B. A., Racine, N., McDonald, S., Tough, S., & Madigan, S. (2021). Child and family factors associated with child mental health and well-being during COVID-19 [Article in Press]. *European Child and Adolescent Psychiatry*. <https://doi.org/10.1007/s00787-021-01849-9>

Medrano, M., Cadenas-Sanchez, C., Oses, M., Arenaza, L., Amasene, M., & Labayen, I. (2021). Changes in lifestyle behaviours during the COVID-19 confinement in Spanish children: A longitudinal analysis from the MUGI project. *Pediatric Obesity*, *16*(4), e12731. <https://doi.org/10.1111/ijpo.12731>

Min, S.-K., Son, W.-H., Choi, B.-H., Lee, H.-J., Ahn, C.-Y., Yoo, J., Park, S., Lee, J.-W., & Jee, Y.-S. (2021). Psychophysical condition of adolescents in coronavirus disease 2019. *Journal of exercise rehabilitation*, *17*(2), 112-119. <https://doi.org/10.12965/jer.2142198.099>

Mitra, R., Waygood, E. O. D., & Fullan, J. (2021). Subjective well-being of Canadian children and youth during the COVID-19 pandemic: The role of the social and physical environment and healthy movement behaviours [Article]. *Preventive Medicine Reports*, *23*. <https://doi.org/10.1016/j.pmedr.2021.101404>

Mzadi, A. E., Zouini, B., Kerekes, N., & Senhaji, M. (2022). Mental Health Profiles in a Sample of Moroccan High School Students: Comparison Before and During the COVID-19 Pandemic [Article]. *Frontiers in Psychiatry*, *12*. <https://doi.org/10.3389/fpsyt.2021.752539>

Ren, H., He, X., Bian, X., Shang, X., & Liu, J. (2021). The Protective Roles of Exercise and Maintenance of Daily Living Routines for Chinese Adolescents During the COVID-19 Quarantine Period [Article]. *Journal of Adolescent Health*, *68*(1), 35-42. <https://doi.org/10.1016/j.jadohealth.2020.09.026>

Tulchin-Francis, K., Stevens, W., Gu, X., Zhang, T., Roberts, H., Keller, J., Dempsey, D., Borchard, J., Jeans, K., & VanPelt, J. (2021). The impact of the coronavirus disease 2019 pandemic on physical activity in U.S. children [Article]. *Journal of Sport and Health Science*, *10*(3), 323-332. <https://doi.org/10.1016/j.jshs.2021.02.005>

Wang, L., Chen, L., Jia, F., Shi, X., Zhang, Y., Li, F., Hao, Y., Hou, Y., Deng, H., Zhang, J., Huang, L., Xie, X., Fang, S., Xu, Q., Xu, L., Guan, H., Wang, W., Shen, J., Li, F., . . . Li, T. (2021). Risk factors and prediction nomogram model for psychosocial and behavioural problems among children and adolescents during the COVID-19 pandemic: A national multicentre study: Risk Factors of Childhood Psychosocial Problems [Article]. *Journal of Affective Disorders*, *294*, 128-136. <https://doi.org/10.1016/j.jad.2021.06.077>

Wunsch, K., Nigg, C., Niessner, C., Schmidt, S. C. E., Oriwol, D., Hanssen-Doose, A., Burchartz, A., Eichsteller, A., Kolb, S., Worth, A., & Woll, A. (2021). The Impact of COVID-19 on the Interrelation of Physical Activity, Screen Time and Health-Related Quality of Life in Children and Adolescents in Germany: Results of the Motorik-Modul Study. *Children*, *8*(2), 1-14. <https://doi.org/10.3390/children8020098>

Zhang, X., Zhu, W., Kang, S., Qiu, L., Lu, Z., & Sun, Y. (2020). Association between physical activity and mood states of children and adolescents in social isolation during the COVID-19 epidemic [Article]. *International Journal of Environmental Research and Public Health*, *17*(20), 1-12. <https://doi.org/10.3390/ijerph17207666>

**Supplementary Table 5. GRADE scores for each outcome.**

| **No. of articles** | **Certainty assessment** | | | | | **Certainty** |
| --- | --- | --- | --- | --- | --- | --- |
|  | Risk of bias | Inconsistency | Indirectness | Imprecision | Publication bias |  |
| Relationship between PA, and psychological and/or behavioral problems | | | | | | |
| 14  ^30,34,40,41,44,45,49,51,56,59,64,65,67,69^ | No | Not serious | Not serious | Not serious | Low | ⊕⊕⊕⊕  High |
| PA | | | | | | |
| 9  ^27,28,32,39,42,43,46,50,54^ | No | Serious^a^ | Not serious | Not serious | Low | ⊕⊕⊕⊕  High |
| Sleep duration | | | | | | |
| 15  ^25,27,28,35,37-39,42,43,46-48,54,57,60^ | No | Serious^a^ | Not serious | Not serious | Low | ⊕⊕⊕⊕  High |
| Sleep Quality | | | | | | |
| 5  ^25,27,28,45,57^ | No | Serious^a^ | Not serious | Serious^b^ | Low | ⊕⊕⊕◯  Moderate |
| QoL | | | | | | |
| 2  ^31, 68^ | No | Serious^a^ | Not serious | Serious^b^ | Low | ⊕⊕⊕◯  Moderate |

Keys

^a^High inconsistency (*I^2^*> 90%)

^b^Small sample size

**Supplementary Table 6. Sensitivity analysis.**

| **Excluding one by one** | | | | |
| --- | --- | --- | --- | --- |
| **Study** | **Odds Ratio** | **Lower Limit** | **Upper Limit** | **P-value** |
| Children | | | | |
| Breidokiené et al. (2021) | -0.217 | -0.257 | -0.176 | 0.000 |
| Chen et al. (2021)_1 | -0.213 | -0.257 | -0.170 | 0.000 |
| Chen et al. (2021)_2 | -0.214 | -0.256 | -0.172 | 0.000 |
| Chen et al. (2021)_3 | -0.217 | -0.259 | -0.174 | 0.000 |
| Chen et al. (2021))_4 | -0.218 | -0.259 | -0.178 | 0.000 |
| Gibert et al. (2021) | -0.212 | -0.252 | -0.171 | 0.000 |
| Ishimoto et al. (2022)_1 | -0.209 | -0.249 | -0.169 | 0.000 |
| Ishimoto et al. (2022)_2 | -0.209 | -0.249 | -0.169 | 0.000 |
| Ishimoto et al. (2022)_3 | -0.213 | -0.253 | -0.172 | 0.000 |
| Ishimoto et al. (2022)_4 | -0.214 | -0.255 | -0.173 | 0.000 |
| Ishimoto et al. (2022)_5 | -0.211 | -0.251 | -0.170 | 0.000 |
| Ishimoto et al. (2022)_6 | -0.217 | -0.257 | -0.177 | 0.000 |
| Ishimoto et al. (2022)_7 | -0.214 | -0.254 | -0.173 | 0.000 |
| Ishimoto et al. (2022)_8 | -0.218 | -0.258 | -0.178 | 0.000 |
| Ishimoto et al. (2022)_9 | -0.214 | -0.254 | -0.173 | 0.000 |
| McArthur et al. (2021)_1 | -0.213 | -0.254 | -0.172 | 0.000 |
| McArthur et al. (2021)_2 | -0.217 | -0.257 | -0.176 | 0.000 |
| Wang et al. (2021)_1 | -0.210 | -0.250 | -0.170 | 0.000 |
| Wang et al. (2021)_2 | -0.204 | -0.243 | -0.165 | 0.000 |
| Zhang et al. (2021)_1 | -0.216 | -0.256 | -0.176 | 0.000 |
| Zhang et al. (2021)_2 | -0.216 | -0.256 | -0.175 | 0.000 |
| Zhang et al. (2021)_3 | -0.213 | -0.254 | -0.173 | 0.000 |
| Zhang et al. (2021)_4 | -0.213 | -0.254 | -0.173 | 0.000 |
| Adolescents | | | | |
| Ghorbani et al. (2021)_1 | -0.209 | -0.248 | -0.169 | 0.000 |
| Ghorbani et al. (2021)_2 | -0.206 | -0.244 | -0.168 | 0.000 |
| Ghorbani et al. (2021)_3 | -0.209 | -0.248 | -0.169 | 0.000 |
| Jackson et al. (2021)_1 | -0.213 | -0.254 | -0.172 | 0.000 |
| Jackson et al. (2021)_2 | -0.217 | -0.258 | -0.177 | 0.000 |
| Jackson et al. (2021)_3 | -0.219 | -0.259 | -0.180 | 0.000 |
| Kang et al. (2021)_1 | -0.215 | -0.256 | -0.174 | 0.000 |
| Kang et al. (2021)_2 | -0.217 | -0.258 | -0.177 | 0.000 |
| Kang et al. (2021)_3 | -0.213 | -0.253 | -0.173 | 0.000 |
| Kang et al. (2021)_4 | -0.214 | -0.254 | -0.173 | 0.000 |
| Laurier et al. (2021)_1 | -0.214 | -0.254 | -0.174 | 0.000 |
| Laurier et al. (2021)_2 | -0.214 | -0.254 | -0.173 | 0.000 |
| Laurier et al. (2021)_3 | -0.214 | -0.254 | -0.174 | 0.000 |
| Laurier et al. (2021)_4 | -0.216 | -0.256 | -0.175 | 0.000 |
| Laurier et al. (2021)_5 | -0.212 | -0.252 | -0.172 | 0.000 |
| Lu et al. (2021)_1 | -0.211 | -0.251 | -0.170 | 0.000 |
| Lu et al. (2021)_2 | -0.213 | -0.254 | -0.172 | 0.000 |
| Lu et al. (2021)_3 | -0.207 | -0.247 | -0.167 | 0.000 |
| Lu et al. (2021)_4 | -0.214 | -0.255 | -0.174 | 0.000 |
| Lu et al. (2021)_5 | -0.218 | -0.258 | -0.178 | 0.000 |
| Lu et al. (2021)_6 | -0.213 | -0.254 | -0.172 | 0.000 |
| Mzadi et. al. (2022)_1 | -0.232 | -0.306 | -0.158 | 0.000 |
| Mzadi et. al. (2022)_2 | -0.229 | -0.301 | -0.156 | 0.000 |
| Ren et al. (2021) | -0.214 | -0.256 | -0.173 | 0.000 |

1. **Abstract video**

The abstract video can be found in the following Figshare: <https://doi.org/10.6084/m9.figshare.20459919.v1>
